# The *PERPETUAL FLOWERING* locus: Necessary but insufficient for genomic prediction of runnerless and other asexual reproduction phenotypes in strawberry

**DOI:** 10.1002/tpg2.70086

**Published:** 2025-08-19

**Authors:** Hillel Brukental, Marta L. Bjornson, Dominique D. A. Pincot, Michael A. Hardigan, Sadikshya Sharma, Nicolas P. Jiménez, Randi A. Famula, Cindy M. Lòpez, Glenn S. Cole, Mitchell J. Feldmann, Steven J. Knapp

**Affiliations:** ^1^ Department of Plant Sciences University of California Davis California USA; ^2^ USDA‐ARS Horticultural Crops Production and Genetic Improvement Corvallis Oregon USA

## Abstract

Strawberry (*Fragaria* × *ananassa*) reproduces sexually through seeds and asexually through stolons. The ability to cost‐effectively clonally propagate hybrid individuals on a large scale has shaped strawberry breeding and production practices. Despite the technical and economic importance of clonal propagation, little is known about the genetic regulation of runnering in strawberry, apart from the pleiotropic effects of *PERPETUAL FLOWERING* (*PF*), a dominant gene introgressed from a wild relative that abolishes temperature‐dependent photoperiod sensitivity and incompletely and variably suppresses runnering. Here, we show that runnering phenotypes are heritable and highly variable in strawberry, ranging from runnerless to prolific in short‐day (*pfpf*) and day‐neutral (*PF*_) plants. The *PF* locus was physically mapped to Mb 26.4–27.3 on chromosome 4B and found to explain 22% of the genetic variance for runnering (78% of the heritability was missing). *PF* was the only runnering‐associated locus identified by genome‐wide association studies among diverse clonal genetic resources and progeny from narrow and wide crosses (1537 individuals). The pleotropic effect of *PF* on runnering was temporal, variable, and incompletely dominant. Genomic selection was found to be a viable strategy for modifying runnering phenotypes in strawberry. Genomic prediction accuracies ranged from 0.53 to 0.79 for runnering, were greater within than between populations, and increased when corrected for *PF*. Our study builds the foundation for improving the productivity of strawberry by developing runnerless cultivars for seed‐propagation or reduced runnering cultivars for clonal‐propagation through phenotypic or genomic selection.

## Core Ideas

- Variation for runnering is substantial and heritable within seasonal and perpetual flowering strawberry populations.
- The pleiotropic suppression of runnering by the *PERPETUAL FLOWERING* (*PF*) allele is continuously variable, temporal, and incompletely dominant.
- The accuracy of breeding value prediction is sufficient for modifying the runner growth characteristics of the strawberry plant by genomic selection.
- The productivity of strawberry can be improved by introducing seed‐propagated cultivars with runnerless and clone‐propagated cultivars with reduced runnering phenotypes.

## Plain Language Summary

The ability to clone individual plants has shaped strawberry breeding and production practices, and runner (stolon) growth is well known to home gardeners and farmers alike and important for the propagation of strawberry cultivars. The energy that the strawberry plant diverts into runner growth can decrease fruit yield. To circumvent maximize fruit yield, farmers mechanically trim runners. The genetic factors that control runner growth are complex but mostly unknown apart from *PERPETUAL FLOWERING*, a gene controlling photoperiod sensitive flowering and affecting runnering. We observed a full spectrum of runner growth phenotypes in seasonal and perpetual flowering populations, found that runnering phenotypes are heritable, and showed that the runner growth of strawberry plants can be modified by phenotypic or genomic selection. Lastly, we identified extreme transgressive segregates for runnering and showed that runnerless and reduced runnering cultivars can be developed to reduce production costs and improve productivity.

**Abbreviations**
ARMS: amplification‐refractory mutation system
DN: day‐neutral
EMM: estimated marginal mean
FS: full sibs
GEBV: genomic‐estimated breeding value
GWAS: genome‐wide association study
LD: linkage disequilibrium
LMM: linear mixed model
*PF*
: *PERPETUAL FLOWERING*
QTL: quantitative trait locus
REML: restricted maximum likelihood
RS: runner score
SD: short‐day
SDP: strawberry diversity panel
SNP: single nucleotide polymorphism
SSR: simple sequence repeat

## 1 INTRODUCTION

Cultivated strawberry (*Fragaria* × *ananassa*), a self‐compatible allo‐octoploid (2*n* = 8*x* = 56), reproduces sexually through either self‐ or cross‐pollination and asexually through stolons (runners) and plantlets (daughter plants) (Heide et al., 2013; Hytönen & Kurokura, 2020; Voth & Bringhurst, 1990). That reproductive flexibility has shaped breeding and cultivar development practices, which rely on the clonal propagation of parents and progeny (Darrow, 1966; Voth & Bringhurst, 1990). Strawberry cultivars are nearly always clonally propagated hybrid (F_1_) individuals originating from crosses between outbred parents with shared ancestry and complex, intertwined pedigrees (Darrow, 1966; Feldmann, Pincot, Seymour, et al., 2024; Hardigan et al., 2020; D. D. A. Pincot et al., 2021).

While breeding has traditionally and necessarily focused on improving fruit quality and production traits in strawberry, adequate runner growth and clone yield (nursery production traits) are necessary for the cost‐effective clonal propagation of cultivars, especially those grown on a large‐scale for annual production systems. Uneconomical clone yields have generally not been a problem for modern short‐day (SD) or day‐neutral (DN) cultivars, which are classified by the absence or presence of flowering under long‐days (14.0–14.9 h), respectively, because most runner sufficiently in nursery production environments; however, some runner weakly, particularly modern DN cultivars purposefully selected for reduced runnering in fruit production environments, for example, the reduced runnering DN cultivar UCD Moxie (US Plant Patent #2019/0380247 P1).

Despite the economic and agricultural importance of asexual reproduction in strawberry, stolon and plantlet growth divert energy and nutrients away from sexual reproduction, alter plant architecture, and are unnecessary and unwanted in fruit‐bearing plants (Heide et al., 2013; Hytönen & Kurokura, 2020; Sønsteby et al., 2021; Voth & Bringhurst, 1990). The runners that emerge in fruit production fields are typically removed by hand to maximize fruit yield, whereas flowers that emerge in bare‐root plant (nursery) production fields are commonly removed by hand to maximize daughter plant (clone) yield. The ideal cultivars for clone‐propagated production systems produce economically viable clone yields in nursery production and an absolute bare minimum of runners in fruit production. That ideal can be difficult to achieve in practice because clone and fruit yield are negatively genetically correlated, although that has not been unequivocally substantiated, partly because runners are normally trimmed in fruit‐bearing plants to negate the pleiotropic effect of runner growth on fruit yield. The ideal cultivars for seed‐propagated production systems are runnerless. Although runnerless phenotypes have long been known in the octoploid, the genetic determinants of runnerless phenotypes are unknown and breeding for runnerless and reduced runnering phenotypes has scarcely been documented (Darrow, 1929, 1966).

Little is known about the genetic regulation of asexual reproduction or the heritability of runnering or clone yield variation within SD and DN populations of octoploid strawberry, apart from the pronounced and well documented temporal pleiotropic effects of the *PERPETUAL FLOWERING* (*PF*) locus on sexual and asexual reproduction (Ahmadi et al., 1990; Bringhurst & Voth, 1976; Bringhurst and Voth, 1980; Bringhurst et al., 1989; Castro et al., 2015; Gaston et al., 2013; Hossain et al., 2019). Those pleiotropic effects are apparent and exposed when progeny of crosses between SD (*pfpf*) and DN (*PF_*) parents are grown under long days (Gaston et al., 2013).

The DN cultivars developed at University of California (UC) Davis (UCD) and many others around the globe are descendants of a hybrid between the SD cultivar Shasta and the *Fragaria virginiana* spp. *glauca* ecotype Wasatch (Ahmadi et al., 1990; Bringhurst and Voth, 1980). That iconic ecotype, collected from the Wasatch Mountains of Utah in 1953 by Royce S. Bringhurst, became the backbone of the UCD DN breeding program (Ahmadi et al., 1990; Bringhurst & Voth, 1976; Bringhurst et al., 1989; Feldmann, Pincot, Cole, et al., 2024; J. Hancock et al., 2008, J. F. Hancock et al., 2018; D. D. A. Pincot et al., 2021). Bringhurst et al. (1989) and Ahmadi et al. (1990) showed that DN descendants of the original hybrid (Shasta × Wasatch) inherited a dominant *PF* allele from Wasatch that is necessary for flowering under long days. The Wasatch *PF* allele, which has been indispensable and widely used in the development of perpetual flowering (DN) cultivars, doubled fruit yields and transformed strawberry production in California (Feldmann, Pincot, Cole, et al., 2024; J. F. Hancock, 2006; J. F. Hancock et al., 2018).

Although SD and DN plants asexually reproduce under a wide range of conditions, runner proliferation and daughter plant growth are weaker in *PF_* than *pfpf* individuals under the long days and high temperatures of summer (Gaston et al., 2013). Using those conditions, Gaston et al. (2013) genetically mapped a quantitative trait locus (QTL) for runnering (*PFRU*) that colocated with the *PF* locus. They showed that phenotypic differences for runnering between *pfpf* and *PF*_ individuals were exposed under long days and likely caused by the pleiotropic effects of the *PF* locus. We are highlighting the distinction here because the *PF* locus was originally identified from the Mendelian distributions of long‐day flowering habit phenotypes (Ahmadi et al., 1990; Bringhurst & Voth, 1976; Bringhurst and Voth, 1980; Bringhurst et al., 1989), whereas the *PFRU* QTL was identified from the continuous distributions of runnering and inflorescence count phenotypes (Gaston et al., 2013).

The effects of *PF* alleles are photoperiod and temperature dependent (Bringhurst & Voth, 1976; Gaston et al., 2013; Heide et al., 2013; Hytönen & Kurokura, 2020; Koskela et al., 2016; Sønsteby & Heide, 2006); nevertheless, across the normal range of temperatures encountered in environments that dominate fruit production, *PFPF* and *PFpf* plants flower continuously, whereas *pfpf* plants cease flowering in the late spring and early summer as temperatures increase and daylengths approach the summer solstice (Ahmadi et al., 1990; Bringhurst & Voth, 1976; Bringhurst et al., 1989; Gaston et al., 2013; Heide et al., 2013; Hytönen & Kurokura, 2020). The higher temperatures over the long days of summer typically stimulate runnering in SD and DN plants (Sønsteby and Heide, 2006).

**Core Ideas**

- Variation for runnering is substantial and heritable within seasonal and perpetual flowering strawberry populations.
- The pleiotropic suppression of runnering by the *PERPETUAL FLOWERING* (*PF*) allele is continuously variable, temporal, and incompletely dominant.
- The accuracy of breeding value prediction is sufficient for modifying the runner growth characteristics of the strawberry plant by genomic selection.
- The productivity of strawberry can be improved by introducing seed‐propagated cultivars with runnerless and clone‐propagated cultivars with reduced runnering phenotypes.

The photoperiod‐dependent inhibition of flowering by the recessive (wildtype) allele in SD (*pfpf*) plants causes a discrete shift from mixed sexual and asexual reproduction to asexual reproduction only, whereas the dominant (mutant) allele (*PF*) knocks out the photoperiod‐dependent inhibition of flowering of the wildtype (Ahmadi et al., 1990; Bringhurst & Voth, 1976; Bringhurst et al., 1989; Gaston et al., 2013). DN (*PF_*) plants flower continuously, exhibit mixed sexual and asexual reproduction over much of their life cycle, and lack the discrete photoperiod‐dependent shift from mixed sexual and asexual reproduction to asexual reproduction only (Sønsteby & Heide, 2006). Sønsteby and Heide (2006) stressed that DN strawberries are obligatory long‐day plants at high temperatures (≥ 27°C), quantitative long‐day plants at intermediate temperatures, and truly DNs only at cooler temperatures (≤ 9°C). The term DN, however, has become deeply ingrained in the lexicons of science and commerce to describe perpetual flowering (*PF*_) plants, and is used throughout this paper for simplicity because temperature‐dependent flowering habit definitions change as temperatures change and because *PF*_ cultivars encounter a wide range of temperatures over an entire growing season in climates where strawberries are produced on a large scale, for example, coastal California.

While the phenotypic and pleiotropic effects of the *PF* locus have been widely discussed (Ahmadi et al., 1990; Bringhurst & Voth, 1976, 1980; Castro et al., 2015; Cockerton et al., 2023; Gaston et al., 2013; Koskela et al., 2016; Lewers et al., 2019; Perrotte et al., 2016; Saiga et al., 2022; Salinas et al., 2017; Verma et al., 2017), the genetic determinants of variation for runnering and clone yield within DN (*PF_*) and SD (*pfpf*) populations have not. Here, we show that the suppression of runnering is highly variable and quantitative among *PF_* plants under long days, that runnering is highly variable within populations of SD (*pfpf*) and DN (*PF_*) plants where the confounding effects of the *PF* locus are obviously absent, and that variation for runnering in SD and DN plants spans the entire range, from runnerless to prolific and unrestrained. The genetic determinants of that phenotypic variation are unknown.

We undertook the studies described here to develop a deeper understanding of the genetics of asexual reproduction and explore genomic prediction as a solution to the problem of accurately predicting runnering phenotypes in octoploid strawberry populations that are either fixed or segregating for *PF* alleles. The heritability of runnering and feasibility of applying genomic selection for runnering has not been previously assessed in strawberry. Our studies were motivated by the prospect of developing highly productive runnerless cultivars for seed‐propagated and near‐runnerless cultivars for clone‐propagated production systems that strike the perfect balance between sexual and asexual reproduction, within the physiological and developmental limits imposed by the biology of the underlying causal genes, including *PF* (Caruana et al., 2018; Gaston et al., 2013; Koskela et al., 2016; Mouhu et al., 2013; Sønsteby and Heide, 2006).

## 2 MATERIALS AND METHODS

### 2.1 Plant material

The plant materials used in our studies included four seed‐propagated F_2_ populations, nine seed‐propagated full‐sib (FS) families, and 932 clonally propagated individuals maintained in the UCD germplasm collection. The latter spanned the range of runnering phenotypes observed in strawberry and were selected to construct a strawberry diversity panel (SDP) for genome‐wide association study (GWAS) and genomic prediction study. The SDP included 15 *Fragaria chiloensis* ecotypes, 24 *F. virginiana* ecotypes, and 893 *Fragaria* × *ananassa* individuals (Supporting Information S1). The *Fragaria* × *ananassa* individuals included 71 UC and 46 non‐UC cultivars developed between 1775 and 2017 and 777 hybrid (F_1_) individuals developed between 1937 and 2021. The origin year, taxa, and pedigrees of these individuals are documented in Supporting Information S1.

We developed nine FS families by crossing elite SD and DN parents with runner scores (RSs) ranging from 2.2 to 4.0 (Table 1). The parents included three cultivars released by UCD in 2023 (UC Surfline, UC Monarch, and UC Keystone) and 11 additional elite F_1_ hybrid individuals selected from FS families developed between 2017 and 2019.

**TABLE 1:** Flowering habit (FH)^a^ and runner score (RS)^b^ phenotypes and A/G single nucleotide polymorphism (SNP) marker (AX‐184947290) genotypes^c^ of the parents of F_1_ individuals and F_2_ and full‐sib families.

			Female parent				Male parent			
Source	Source ID	n	Parent ID	FH	SNP	RS	Parent ID	FH	SNP	RS
F_1_	19A907P022	1	17C321P015	SD	AG	5.0	Royal Royce	DN	AG	2.8
	19A907P024	1	17C321P015	SD	AG	5.0	Royal Royce	DN	AG	2.8
	19A908P012	1	17C321P015	SD	AG	5.0	55C032P001	DN	AG	1.3
	19A908P053	1	17C321P015	SD	AG	5.0	55C032P001	DN	AG	1.3
F_2_	21S950	48	19A907P022	—	AA	1.3	19A907P022	—	AA	1.3
	21S951	135	19A907P024	—	AG	1.3	19A907P024	—	AG	1.3
	21S952	48	19A908P012	—	AG	1.0	19A908P012	—	AG	1.0
	21S953	43	19A908P053	—	AG	1.0	19A908P053	—	AG	1.0
Full‐sib	22C315	46	UC Surfline	SD	GG	3.7	19B091P078	DN	AG	3.2
	22C324	37	UC Monarch	SD	GG	3.8	19B091P078	DN	AG	3.2
	22C339	44	17C618P658	SD	GG	3.2	19B091P078	DN	AG	3.2
	22C346	49	18C430P006	SD	GG	4.0	19B033P006	DN	GG	3.0
	22C409	49	UC Keystone	DN	AG	3.2	19B033P006	DN	GG	3.0
	22C410	27	UC Keystone	DN	AG	3.2	18B061P100	DN	AG	2.2
	22C426	48	17C121P097	DN	AG	2.4	19C174P009	SD	GG	2.6
	22C485	46	19C155P035	DN	AG	3.6	19B091P078	DN	AG	3.2
	22C498	26	18B061P005	DN	AG	2.5	17C693P610	SD	GG	4.0

^a^ The flowering habits of the parents were classified as short‐day (SD) or day‐neutral (DN) from the absence or presence of flowering, respectively, under long days (13.5–14.5 h) in Winters, CA.

^b^ The runner scores of the parents were recorded using an ordinal scale, where 1 = *runnerless*, 2 = *weak runnering*, 3 = *intermediate runnering*, 4 = *strong runnering*, and 5 = *extreme runnering* (see Figure 3A).

^c^ AX‐184947290 is an A/G SNP on a 50,000 Axiom array (Hardigan et al., 2020). AX‐184947290 is associated with the *PF* locus and highly predictive of *PF* genotypes in short‐day and day‐neutral cultivars developed at University of California, Davis, between 1935 and 2012. The physical location of AX‐184947290 on chromosome 4B in the UCD Royal Royce genome is by 27,294,311 (https://phytozome‐next.jgi.doe.gov/info/FxananassaRoyalRoyce_v1_0).

F_2_ populations (21S950, 21S951, 21S952, and 21S953) were developed by self‐pollinating four runnerless to near‐runnerless F_1_ individuals (19A907P022, 19A907P024, 19A908P012, and 19A908P053) developed from crosses between parents with contrasting runnering phenotypes (Table 1). The crosses were 17C321P015 × Royal Royce and 17C321P015 × 55C032P001. We selected two runnerless F_1_ individuals from each FS family: 19A907P022 and 19A907P024 from 17C321P015 × Royal Royce and 19A908P012 and 19A908P053 from 17C321P015 × 55C032P001 (Table 1). The selected individuals were manually self‐pollinated over the winter of 2020–2021 to produce seeds of two 17C321P015 × Royal Royce F_2_ populations (21S950 and 21S951) and two 17C321P015 × 55C032P001 F_2_ populations (21S952 and 21S953).

17C321P015 is an SD individual (*pfpf*) with an aggressive runnering phenotype (RS = 5.0) developed from a cross between Cabrillo and PI551727. PI551727 (CA 1234 Mendocino) is an SD *F. chiloensis* spp. *lucida* ecotype with an aggressive runnering phenotype (RS = 5.0) typical of ecotypes of this species. Cabrillo is a DN cultivar (*PFpf*) with an intermediate runnering phenotype (RS = 3.0) typical of modern DN cultivars. Royal Royce is a DN cultivar (*PFpf*) with slightly weaker runnering (RS = 2.8) than Cabrillo. 55C032P001 is a runnerless (RS = 1.3), DN individual developed by Royce S. Bringhurst in 1955 from a cross between the SD cultivar Shasta (*pfpf*; RS = 3.0) and Wasatch, an *F. virginiana* spp. *glauca* ecotype. Wasatch is the source of the dominant *PF* allele that Bringhurst and Voth (1980) introgressed into SD genetic backgrounds, starting with Shasta (Ahmadi et al. 1990).

### 2.2 Field experiments and phenotyping

SDP individuals were grown and clonally propagated annually by rooting plantlets in a field nursery in Winters, CA, over several years (2017–2023, excluding 2018). Our study location was the same as that originally used by Ahmadi et al. (1990) to discover the *PF* locus. The annual propagation cycle started with planting two or three bare‐root plants/individual in the field in April and ended with the harvest of rooted plantlets (bare‐root plants) in December or January. The row spacing was 1.0 m with 0.3 m between plots. Runners were trimmed, if necessary, to prevent cross‐contamination between plots. The bare‐root plants were dug from the field in December or January of each year, cleaned, trimmed, and stored at 4°for 6–10 weeks before being replanted to repeat the annual propagation cycle.

Over the course of nursery propagation cycle each year, SDP individuals were phenotyped for photoperiod‐dependent flowering habit and scored for runnering. The flowering habits of individuals were classified as SD or DN by the absence or presence of flowering, respectively, under long daylengths (13.5–14.5 h) in late spring and early fall. SDP individuals were phenotyped for runnering in August of each year using ordinal RSs on a 1–5 scale, where 1 = *runnerless*, 2 = *weak runnering*, 3 = *intermediate runnering*, 4 = *strong runnering*, and 5 = *extreme runnering*. The RSs compiled in Supporting Information S1 are estimated marginal means (EMMs) calculated from phenotypes observed over multiple years in Winters, CA. The number of observations per individual ranged from 1 to 6 (the harmonic mean number of observations per individual was 1.92).

We grew approximately 100 seed‐propagated individuals from the 17C321P015 × Royal Royce and 17C321P015 × 55C032P001 FS families in a Winters, CA, field experiment in 2018–2019. Seedlings of those individuals were grown in a shade house over the summer of 2018, transplanted to the field in September 2018, and phenotyped for runnering in June 2019. Two runnerless individuals were identified and selected within each FS family. Those F_1_ individuals were transplanted from the field to a greenhouse in July 2019 and self‐pollinated over the winter of 2019–2020 to create four F_2_ populations (Table 1).

F_2_ progeny (274 individuals from four families) were phenotyped for runner count in a Davis, CA, field experiment in 2021–2022 (Table 1). F_2_ seeds were germinated in June 2021; seedlings were transplanted to the field in September 2021; and plants were phenotyped by counting primary, secondary, and tertiary runners on March 26, April 9 and 22, May 6 and 20, and June 3 and 16, 2022. The photoperiods on those dates were 12.3, 13.0, 13.3, 14.1, 14.3, 14.4, and 14.5 h, respectively. The photoperiod on the summer solstice (June 21, 2022) was 14.5 h.

FS progeny (372 individuals from nine families) were phenotyped for RS and count in a Davis, CA, field experiment in 2023‐2024. FS seeds were germinated June 2023, seedlings were transplanted to the field in September 2023, and plants were phenotyped by counting inflorescence number and primary runner number on May 1, June 1, and July 1, 2024. The photoperiods on those dates were 13.5, 14.4, and 14.5 h, respectively. FS progeny were scored for runnering as described for the SDP population on July 1, 2024.

### 2.3 Genotyping

Single nucleotide polymorphism (SNP) genotypes for the SDP population were obtained from previous studies (Feldmann, Pincot, Seymour, et al., 2024; Hardigan et al., 2021; D. D. Pincot et al., 2022). DNA samples of SDP individuals were previously genotyped with either 50,000 or 850,000 Axiom SNP arrays designed by Hardigan et al. (2020) and developed in collaboration with the ThermoFisher Affymetrix Expert Design Program (https://www.thermofisher.com/order/catalog/product/551041). Freshly emerged leaves were harvested from F_2_ and FS individuals, lyophilized, and powdered for DNA isolation using the E‐Z 96 Plant DNA Kit (Omega Bio‐Tek) as described by Knapp et al. (2024). DNA samples of these individuals were genotyped with the 50,000 Axiom SNP array Hardigan et al. (2020).

The probe DNA sequences for SNPs on the 50,000 and 850,000 Axiom arrays used in our studies were physically anchored to the haplotype‐phased UCD Royal Royce genome in silico (FaRR1; https://phytozome‐next.jgi.doe.gov/info/FxananassaRoyalRoyce_v1_0) and unphased ‘Camarosa’ genome in silico (FaCA1; Edger et al. (2019); https://phytozome‐next.jgi.doe.gov/info/Fxananassa_v1_0_a1). The physical positions of SNPs in the UCD Royal Royce genome are reported throughout this paper. DNA sequences for the SNP probes and physical coordinates are compiled in Supporting Information S2 for both genomes using the chromosome nomenclature described by Hardigan et al. (2020). That nomenclature is cross‐referenced to other linkage group and chromosome nomenclatures in Supporting Information S3. Homoeologous chromosomes in the UCD Royal Royce genome were numbered 1–7 and oriented according to the rules used for *Fragaria vesca*, the A‐genome ancestor (Edger et al., 2018; Shulaev et al., 2011). As previously described (Hardigan et al., 2020), that was feasible because of synteny among homoeologous A, B, C, and D genome chromosomes. DNA samples that passed quality and quantity control standards were genotyped on a GeneTitan HT Microarray System by ThermoFisher Scientific. SNP genotypes were called using the Affymetrix Axiom Analysis Suite (v1.1.1.66, Affymetrix).

SNP genotypes were curated and filtered for GWAS and other analyses by retaining those with call rates ≥ 89% and in silico assigned physical positions corroborated by de novo genetic mapping (the latter are identified in Supporting Information S2). The genomic relationship matrix was estimated among SDP, F_2_, and FS individuals using the *rrBLUP::A.mat()* function in the R package “rrBLUP” with a minor allele frequency cutoff of 0.05 (min.MAF = 0.05), a maximum missing data cutoff of 0.8 (max.missing = 0.8), and imputation of missing data (return.imputed = TRUE) (D. D. A. Pincot et al., 2020). After data processing, 41,794 SNPs were retained for analyses of F_2_ and FS populations, whereas 42,903 SNPs were retained for analyses of the SDP population. We genotyped 194 SDP individuals with the 850,000 Axiom SNP array (Hardigan et al., 2020). After data processing, 799,895 were retained for analyses of those individuals, whereas 625,039 were retained for analyses of the subset of 66 UC individuals (Supporting Information S2).

Genetic relationships among SDP, F_2_, and FS individuals were visualized by constructing a neighbor‐joining tree using the *nj()* function in the “ape” R package (Paradis et al., 2004) and drawing the tree using the R package “ggtree” (Yu et al., 2017). The Euclidean distance matrix for this analyses was estimated from 49,483 array‐genotyped SNPs using the *dist()* function in the R “stats” package (R Core Team, 2013).

### 2.4 Statistical analyses

The RSs observed among SDP individuals were analyzed using the lmer() linear mixed model (LMM) function in the R package “lme4” (Bates et al. 2015; https://cran.r‐project.org/web/packages/lme4/index.html), The LMM for that analysis was as follows:

(1) $$y_{ij}=G_{i}+Y_{i}j+{GY}_{ij}$$

where $y_{ij}$ is the RS for the ith individual in the jth year, $G_{i}$ is the fixed effect of the *i*th individual, and $Y_{j}$ is the random effect of the jth year, and $GY_{ij}$ is the random effect of the interaction between individual and year. A single RS was recorded each year, the composition of the germplasm collection changed year to year, and the number of years per individuals ranged from one to six. The harmonic mean number of replications per individual was 1.92. EMMs for RS were estimated using the R package “emmeans” (Lenth, 2019).

The individual, year, and individual × year variance components and broad‐sense heritability on a clone‐mean basis ($H^{2}=V_{G}(V_{G}+V_{GY}/r$) were estimated for RS using restricted maximum likelihood (REML) in the R package “lme4” (Bates et al. 2015, where $V_{G}$ is the REML estimate of the individual variance component, $V_{GY}$ is the REML estimate of the individual × year variance component, and h=1.92 is the harmonic mean of the number of observations per individual, calculated using *pysch::harmonic.mean()* (Revelle, 2021).

Genomic‐estimated narrow‐sense heritability ($h^{2}$) was estimated for the SDP, F_2_, and FS populations using the *rrBLUP:: mixed.solve()* function, where $h^{2}=V_{A}/(V_{A}+V_{GY)}/r$) for the SDP population, $h^{2}=V_{A}/(V_{P}$ for the F_2_ and FS populations, $V_{A}$ is the genomic‐estimated additive genetic variance, and $V_{P}$ is the phenotypic variance on an individual‐plant basis.

Several statistics were estimated for the *PF*‐associated SNP AX‐184947290 using the LMMs described above. The additive and dominance effects of AX‐184947290 were estimated by $\hat{a}=\left({\bar{y}}_{\text{AA}}-{\bar{y}}_{\text{GG}}\right)/2$ and $\hat{d}={\bar{y}}_{\text{AG}}-\left({\bar{y}}_{\text{AA}}+{\bar{y}}_{\text{GG}}\right)/2$, respectively, where ${\bar{y}}_{\text{AA}}$, ${\bar{y}}_{\text{AG}}$, and ${\bar{y}}_{\text{GG}}$ are the respective phenotypic means for AA, AG, and GG SNP genotype. The degree of dominance of AX‐184947290 was estimated by |$\hat{d}/\hat{a}$| (Falconer, 1996; Walsh, 2001). The percentage of the genetic variance explained by AX‐184947290 was estimated by $\text{GVE}={\hat{\sigma }}_{M}^{2}/{\hat{\sigma }}_{A}^{2}$, where ${\hat{\sigma }}_{M}^{2}$ is the bias‐corrected average semivariance REML estimate of the variance associated with AX‐184947290 (Feldmann et al., 2021, 2022). The percentage of the phenotypic variance explained by AX‐184947290 was estimated by $\text{PVE}={\hat{\sigma }}_{M}^{2}/{\hat{\sigma }}_{P}^{2}$.

### 2.5 Genome‐wide association study

GWAS analyses were applied to the SDP population for flowering habit and RS, F_2_ population for runner count, and FS population for RS, runner count, and inflorescence count using GEMMA v0.98.1 (Zhou & Stephens, 2012). The RS EMMs and binary flowering habit classification (SD and DN) were used as input for GWAS of the SDP population. The runner count, inflorescence count, and RS observations for individual time points were used as input for GWAS of F_2_ and FS populations. The GEMMA ‘lmm2’ function and kinship (K) matrix were used to correct for the population structure (Zhou & Stephens, 2012). The *p*‐values were adjusted for a false discovery rate (FDR) of 0.05 and reported as $-\log_{10}\left(\text{FDR p-value}\right)$ score. Three lead *PF*‐associated SNPs (AX‐184947290, AX‐184219235, and AX‐184208850) were used as covariates in GEMMA to search for associations between genetic variants and phenotypes. We tested AX‐184947290 and AX‐184219235 individually; AX‐184947290 and AX‐184219235 in combination; and AX‐184947290, AX‐184219235, and AX‐184208850 in combination. Lastly, we used the *GWAS()* function of the “rrBLUP” R package for GWAS of SDP individuals genotyped with the 850,000 SNP array using flowering behavior coded as 0 (SD) or 1 (DN). The kinship matrix was estimated using the *rrBLUP::A.mat()* function and incorporated in the “rrBLUP” analysis to control for population structure.

### 2.6 Haplotype analyses

The linkage phases of 50,000 array‐genotyped SNPs were unknown among individuals in the SDP and F_2_ populations. They were inferred in the latter by genetic mapping and by comparing haplotypes inferred among SDP individuals. We used Beagle 5.4v (Browning et al., 2021) with default parameters to infer the haplotypes of 51 SNPs that were associated with the *PF* locus and had minor allele frequencies (MAFs) ≥ 0.3 in the SDP population. GWAS‐estimated SNP effects with FDR‐corrected $-\log_{10}\left(\text{p-value}\right)>2$ for flowering habit or RS were selected for phasing. This filtering process yielded 51 SNPs spanning bp 26,529,027 to 29,635,684 on chromosome 4B. The squared correlation coefficient linkage disequilibrium (LD) statistic ($R^{2}$) was estimated among the 51 SNPs using the R package “snpStats,” version 1.58.0 (https://bioconductor.org/packages/snpStats).

### 2.7 Genomic prediction analyses

Genomic relationship matrices (G) and genomic‐estimated breeding values (GEBVs) were estimated by genomic best linear unbiased prediction (G‐BLUP) for RS in the SDP population, runner count in the F_2_ population, and runner count and inflorescence count in the FS population using the *mixed.solve()* function in the R package rrBLUP (Endelman, 2011; Endelman & Jannink, 2012). GEBVs were estimated with and without incorporating the AX‐184947290 SNP as a fixed effect in the G‐BLUP analysis. Genomic predictive ability ($r_{\hat{a},\bar{y}}$) and prediction accuracy ($r_{\hat{a},\bar{y}}/h$) were count within and between strawberry populations by cross‐validation, where $\hat{a}$ is the GEBV and $\bar{y}$ is the EMM. We used the SDP population (n=932) and subset of UC individuals (n=672) in the SDP population (SDP‐UC) for cross‐validation. Cross‐validation was performed with 1000 Monte Carlo iterations within the SDP, SDP‐UC, and FS populations; between the SDP and FS; and SDP‐UC and FC populations by randomly splitting individuals into training (80%) and validation (20%) subsets (Burman, 1989; Molinaro et al., 2005; Poland & Rutkoski, 2016).

## 3 RESULTS

### 3.1 Variation for runnering encompasses the entire phenotypic range, from runnerless to prolific

**FIGURE 1:**
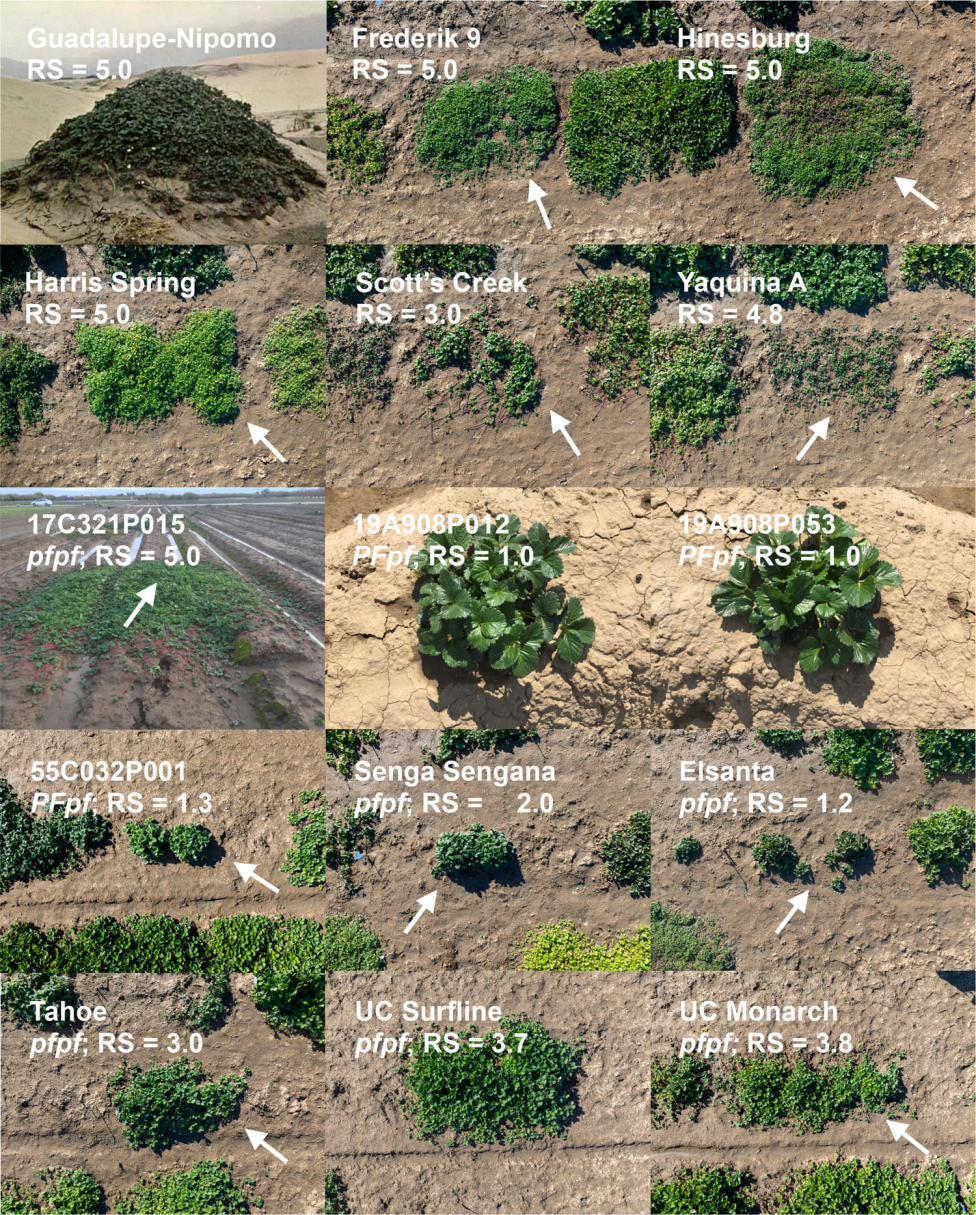
Variation for runnering in octoploid strawberry. Unless otherwise noted, the phenotypes shown in these photographs (shot to scale by Fred Greaves for University of California, Davis) were observed on November 11, 2024, Winters, CA. Guadalupe‐Nipomo, an *F. chiloensis* spp. *lucida* ecotype carpeting a sand dune near Oso Flaco Beach, CA (August 1963). Frederik 9 and Hinesburg are *F. virginiana* spp. *virginiana* ecotypes. Harris Spring and Scott's Creek are *F. virginiana* spp. *platypetala* ecotypes. Yaquina A is an *F. chiloensis* spp. *pacifica* ecotype. 19A908P012 and 19A908P053 are runnerless 17C321P105 × 55C032P001 hybrids (July 2020, Winters, CA). 17C321P015 is a hybrid between Cabrillo and PI551727, an *F. chiloensis* spp. *lucida* ecotype (December 2017, Winters, CA). 55C032P001 is a runnerless day‐neutral hybrid. Senga Sengana, Elsanta, Tahoe, UC Surfline, and UC Monarch are short‐day cultivars. *PF* genotypes were predicted from the segregation of *PF* in full‐sib families and by using a single nucleotide polymorphism (SNP) marker (AX‐184947290) associated with the *PF* locus. RS is the estimated marginal mean for RS over years. Arrows identify the labeled individuals where unrelated individuals appear in growth boundaries.

**FIGURE 2:**
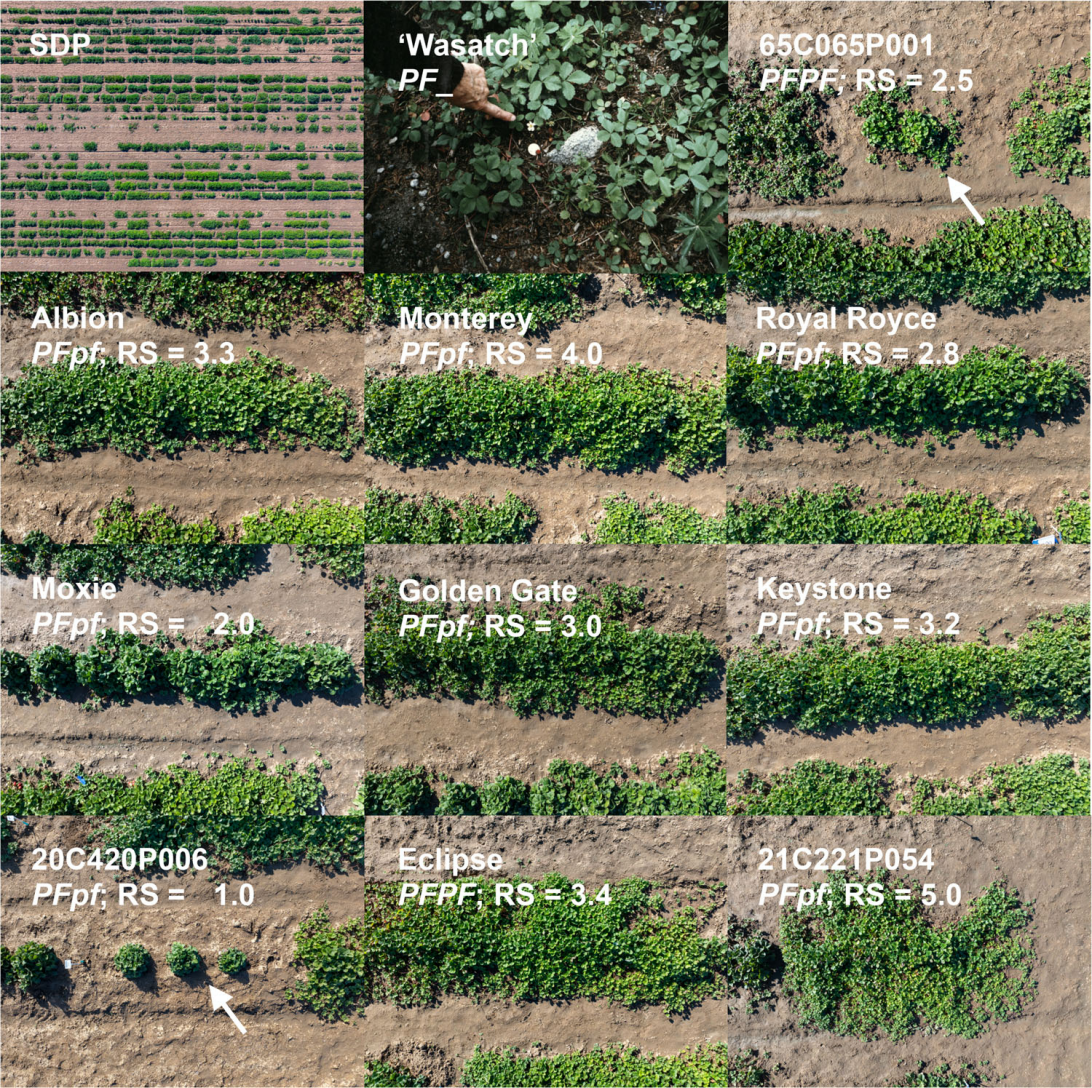
Variation for runnering among day‐neutral (*PFPF* and *PFpf*) individuals known to be descendants of Wasatch, the *F. virginiana* spp. *glauca* donor of the *PF* allele. The Wasatch photograph shows the original *PF* donor plant collected in July, 1953, from the Wasatch Mountains of Utah (reproduced from an original photograph in the Royce S. Bringhurst Special Collection [UUS COLL MSS 515], Merrill‐Cazier Library, Utah State University; https://library.usu.edu/archives/). The phenotypes shown in the other photographs (shot to scale by Fred Greaves for University of California (UC), Davis) were observed on November 11, 2024 in Winters, CA. *PF* genotypes were predicted using a single nucleotide polymorphism (SNP) marker (AX‐184947290) associated with the *PF* locus. RS is the estimated marginal mean for runner score over years. Arrows identify the labeled individuals where unrelated individuals appear in growth boundaries. The aerial photograph (upper left corner) displays a subset of the strawberry diversity panel (SDP) and other individuals preserved in the clonal germplasm collection at UC Davis.

**FIGURE 3:**
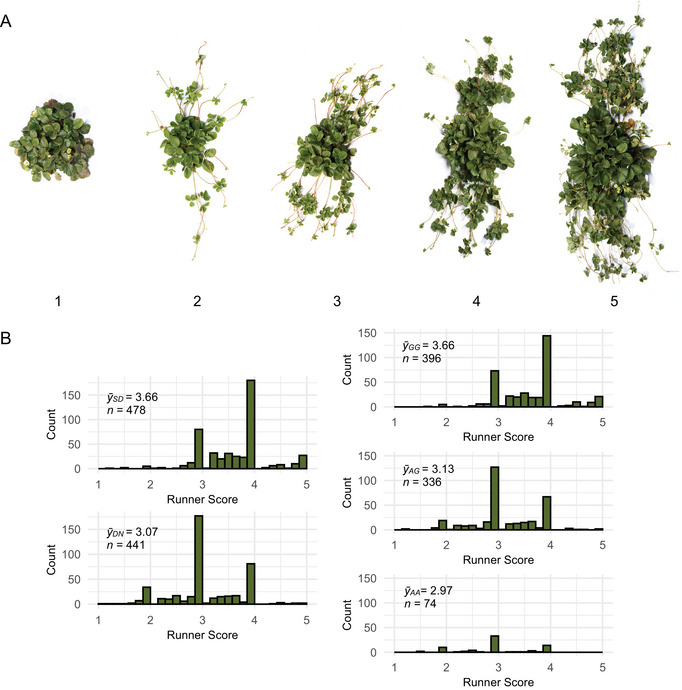
Runner score distributions of strawberry diversity panel individuals classified as short‐day (SD = *pfpf*; n=479) or day‐neutral (DN = *PF*_; n=441) over six growing seasons in Winters, CA. (A) Archetypal phenotypes of individuals scored for runnering on an ordinal scale, where 1 = *runnerless*, 2 = *weak runnering*, 3 = *intermediate runnering*, 4 = *strong runnering*, and 5 = *extreme runnering* (shot to scale by Fred Greaves for University of California, Davis, July 2024, Winters, CA). (B) Histograms were plotted using the phenotypic means (estimated marginal means (EMMs) = $\bar{y}$) for runner scores over years (the harmonic mean number of observations per individual was 1.92). The $\bar{y}$ distributions of strawberry diversity panel (SDP) individuals classified as SD or DN are shown in the left column with their group means (${\bar{y}}_{\text{SD}}$ and ${\bar{y}}_{\text{DN}}$). The $\bar{y}$ distributions of SDP individuals with GG, AG, and AA AX‐184947290 single nucleotide polymorphism (SNP) genotypes are shown in the right column of histograms with their genotypic means (${\bar{y}}_{\text{GG}}$, ${\bar{y}}_{\text{AG}}$, and ${\bar{y}}_{\text{AA}}$).

Figures 1, 2, 3 depict the range of stolon (runner) and plantlet (daughter plant) growth phenotypes observed among several thousand seed‐propagated FS progeny in early‐stage selection nurseries and 3000 clonally propagated SD and DN individuals observed in the UC strawberry germplasm collection over a period of 9 years (2015–2023). That variation was captured by the sample of 932 clonally propagated SDP individuals phenotyped for photoperiod‐dependent flowering habit and runnering in a low‐elevation nursery production environment (Winters, CA; 38.52°N, 121.97°W; 41 m) where photoperiods ranged from 9.5 h to 14.9 h over the March to December growing season (Figure 3; Supporting InformationS1). The term “runnering” applied here and throughout our paper, though informal, is commonly used in research and commerce to describe stolon (runner) and plantlet (daughter plant) growth phenotypes.

**FIGURE 4:**
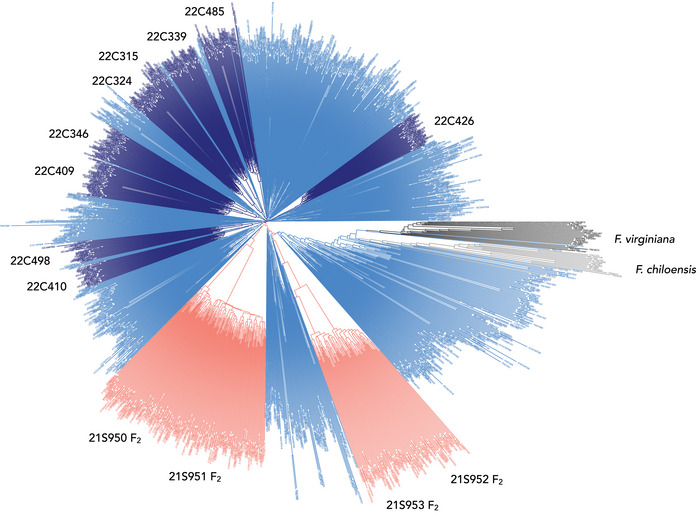
Genetic relationships among 932 strawberry diversity panel, 270 F_2_, and 372 full‐sib individuals genotyped with an Axiom 50,000 single nucleotide polymorphism (SNP) array. Genetic relationships were estimated from 49,483 SNPs. Clades identified by 21C950 and 21C951 are 17C321P015 × UCD Royal Royce F_2_ families. Clades identified by 21C952 and 21C953 are 17C321P015 × 55C032P001 F_2_ families. Clades identified by 22C numbers are full‐sib families developed from crosses between elite short‐day and day‐neutral individuals between 2017 and 2019 at University of California, Davis. The other branches and nodes identify strawberry diversity panel individuals, including *F. chiloensis* and *F. virginiana* ecotypes.

The SDP included 15 *F. chiloensis* ecotypes, 24 *F. virginiana* ecotypes, and 893 *Fragaria* × *ananassa* individuals with runner and daughter plant growth (runnering) phenotypes spanning the range found in the octoploid, from runnerless to prolific and unrestrained (Figures 1, 2, 3; Supporting Information S1). Genetic relationships among these individuals are depicted in Figure 4, along with individuals from two exotic × exotic (17C321P015 × 55C032P001) F_2_ families, two elite × exotic (17C321P015 × UCD Royal Royce) F_2_ families, and nine elite × elite FS families that were phenotyped for runnering in our study (Table 1; Supporting Information S4 and S5). The nine FS families were developed from crosses among 13 elite UC parents (Table 1).

We knew from previous studies that we needed to correct for the photoperiod‐dependent pleiotropic effects of the dominant Wasatch *PF* allele to identify other loci affecting runnering phenotypes (Ahmadi et al., 1990; Gaston et al., 2013). SDP individuals were classified as SD or DN by the absence or presence of flowering under long‐days (14.0–14.9 h), respectively (Figure 3B; Supporting Information S1). The repeatability of our flowering habit classifications was 99% over six growing seasons (March–December) in Winters, CA (38.52°N). SD individuals were inferred to be homozygous recessive (*pfpf*), whereas DN individuals were inferred to be heterozygous or homozygous dominant (*PF*_) for *PF* alleles (Ahmadi et al., 1990; Bringhurst & Voth, 1976; Bringhurst et al., 1989; Gaston et al., 2013; Perrotte et al., 2016).

SDP individuals were phenotyped for runnering in our clonal propagation nursery in Winters, CA, over six growing seasons (March–December) using an ordinal RS scale, where 1 = *runnerless*, 2 = *weak runnering*, 3 = *intermediate runnering*, 4 = *strong runnering*, and 5 = *extreme runnering* (Figure 3). The number of years of observation ranged from one to six per individual (the harmonic mean number of years of observation was 1.92). To complement our analyses of full‐season phenotypes of clonally propagated SDP individuals, seed‐propagated exotic × exotic (17C321P015 × 55C032P001) F_2_, elite × exotic (17C321P015 × UCD Royal Royce) F_2_, and elite × elite FS progeny were phenotyped by counting runners under long days (13.0–14.5 h photoperiods in Davis and Winters, CA). The runner count phenotypes of F_2_ individuals were recorded on seven dates with photoperiods ranging from 13.0 to 14.5 h (Supporting Information S4). Our experience in the F_2_ study guided the phenotyping schedule applied in the FS study. FS individuals were phenotyped on three dates (May 1, June 1, and July 1) with photoperiods ranging from 13.5 to 14.5 h (Supporting Information S5). Unless otherwise noted, we are reporting statistics estimated from phenotypes observed on the longest days when phenotypic differences and the temporal effect of the *PF* locus were the greatest.

The individuals in our study populations sampled genetic variation across the entire diversity range, from exotic (ecotypes of the wild relatives) to elite (modern UC cultivars). Among SDP individuals, 478 were classified as SD and 441 were classified as DN (Figure 3B; Supporting Information S1). The *Fragaria* × *ananassa* individuals included 80 SD cultivars, 39 DN cultivars, 776 UC cultivars, and other hybrids developed between 1969 and 2021 (the SDP‐UC training population), and 691 UC cultivars and other hybrids developed between 2016 and 2021. The 893 *Fragaria* × *ananassa* individuals selected for the highly admixed SDP population originated from 554 unique pedigrees (FS families) among 489 parents (Figure 4; Supporting Information S1). The runnering phenotypes shown in Figures 1 and 2, with five exceptions (Guadalupe‐Nipomo, 17C321P015, 19A908P012, 19A908P053, and Wasatch), were observed on November 11, 2024 after a full‐season of runner growth and clonal propagation in Winters, CA, and were shot to scale to ensure accurate visual comparisons (the mother plants were transplanted March 2024). The runners of those individuals were periodically trimmed to prevent cross‐contamination between individuals; hence, the phenotypes shown for wild relatives and other individuals with prolific and unrestrained runnering are less extreme than would have been observed without runner trimming.

Untrimmed phenotypes are shown in Figure 1 for an *F. chiloensis* spp. *lucida* ecotype (Guadalupe‐Nipomo) in situ and 17C321P015 ex situ, a hybrid between the UC DN cultivar Cabrillo and an *F. chiloensis* spp. *lucida* ecotype (PI551727) native to Cape Mendocino, CA. The iconic photograph of the Guadalupe‐Nipomo ecotype, captured by Royce S. Bringhurst near Oso Flaco Beach, CA, illustrates the coastal adaptation and prolific and unrestrained runner growth of this wild relative in a sand dune habitat (Bringhurst et al., 1977). The phenotype shown for 17C321P015 depicts a single season of nursery growth from a single mother plant (identified by the arrow) without runner trimming (Figure 1). The diameter of the labyrinth of runners and daughter plants emanating from the 17C321P015 mother plant was approximately 3.7 m at the end of the clonal propagation season (December 2017).

At the opposite extreme are the runnerless to near‐runnerless phenotypes of several DN Wasatch descendants, for example, 55C032P001 (55.32‐1), 20C420P006, and UCD Moxie (Figures 1 and 2). 55C032P001 is a first‐generation, DN descendant of Shasta × Wasatch and donor of the *PF* allele found in 65C065P601, Aptos, Brighton, and Hecker, the earliest UC DN cultivars (Ahmadi et al., 1990; Bringhurst and Voth, 1980; D. D. A. Pincot et al., 2021). 65C065P601 (65.65‐601) is a third‐generation DN descendant of Shasta × Wasatch, parent of the DN cultivar Brighton, and grandparent of the DN cultivar Selva (Ahmadi et al., 1990; Bringhurst and Voth, 1980). High‐density SNP haplotypes for those individuals facilitated analyses to pinpoint the physical location of the *PF* locus.

### 3.2 The runnering phenotypes of strawberry are highly heritable and stable

We found runnering phenotypes to be highly heritable, particularly among clonally propagated plants (Table 2). The broad‐sense heritability on a clone‐mean basis ($H^{2}$) for RS was 0.86 in the SDP population (Table 2). Our estimates of narrow‐sense heritability ($h^{2}$) for RS or count in the SDP, F_2_, and FS populations were more moderate, ranging from 0.37 to 0.60 (Table 2). Several transgressive phenotypes were recovered in our breeding and genetic studies, for example, the runnerless phenotypes of the F_1_ parents of our 17C321P015 × Royal Royce F_2_ populations (19A907P022 and 19A907P024) were transgressive (Table 1; Figures 1, 2, 3). The runner scores for both were 1.3, whereas the RSs of the parents were 2.8 and 5.0. Conversely, the runnerless to near‐runnerless phenotypes recovered among 17C321P015 × 55C032P001 progeny (FS) were anticipated because the 55C032P001 parent was runnerless (RS = 1.3). The runnerless phenotype of 55C032P001 was unaffected by temperature and photoperiod variation over nine field growing seasons (March–December) in Winters, CA (Figure 1). Similarly, the phenotypes of 20C420P005 and the other runnerless to near‐runnerless individuals identified in our studies have been stable across years and nursery and fruit production environments (Figures 1 and 2; Supporting Information S1).

**TABLE 2:** Additive genetic variance ($V_{A}$), coefficient of additive genetic variance (${\text{CV}}_{A}$), and heritability of runnering phenotypes in strawberry.

Population^a^	Trait^b^	n	$y_{\text{MIN}}$	$y_{\text{MAX}}$	$V_{A}$	${\text{CV}}_{A}$	$h^{2}$	$H^{2}$
Diversity panel	Runner score	932	1	5	0.3	15.9	0.60	0.86
F_2_ families	Runner count	270	0	104	58.5	32.7	0.37	—
Full‐sib families	Runner count	372	4	98	183.9	30.7	0.53	—
	Inflorescence count		0	41	19.1	177.7	0.60	—

^a^ Statistics were estimated from clonal replicates of strawberry diversity panel (SDP) individuals, unreplicated seed‐propagated 17C321P015 × Royal Royce and 17C321P015 × 55C032P001 F_2_ progeny, and unreplicated seed‐propagated full‐sib progeny from nine crosses between elite parents with contrasting runnering phenotypes (Table 1). n is the number of individuals in the population. $y_{\text{MIN}}$ and $y_{\text{MAX}}$ are phenotypic minimums and maximums, respectively. Genetic variance among clones of individuals ($V_{G}$) and broad‐sense heritability on a clone‐mean basis ($H^{2}=V_{G}/V_{\bar{P}}$) were estimated for runner score among SDP individuals, where $V_{\bar{P}}$ is the phenotypic variance on a clone‐mean basis. Genomic additive genetic variance ($V_{A}$) and narrow‐sense heritability on a clone‐mean basis ($h^{2}=V_{A}/V_{\bar{P}}$) were estimated for runner score among SDP individuals. $V_{A}$ and narrow‐sense heritability on a individual‐plant basis ($h^{2}=V_{A}/V_{P}$) were estimated for runner count among F_2_ progeny and runner and inflorescence count among full‐sib progeny, where $V_{P}$ is the phenotypic variance on an individual plant basis. ${\text{CV}}_{A}=\sqrt{V_{A}}/\bar{y}\times 100$, where $\bar{y}$ is the population mean.

^b^ The flowering habits of single nucleotide polymorphism (SNP) individuals were classified as short‐day or day‐neutral from the absence or presence of flowering, respectively, under long days (13.5–14.5 h) over a period of 6 years in Winters, CA. The runner scores of SDP individuals were similarly recorded over a period 6 years in Winters, CA using an ordinal scale, where 1 = *runnerless*, 2 = *weak runnering*, 3 = *intermediate runnering*, 4 = *strong runnering*, and 5 = *extreme runnering* (see Figure 3A). F_2_ and full‐sib progeny were phenotyped by counting the number of primary runners observed in Davis or Winters, CA, respectively, within 1 week of the summer solstice (the daylength was 14.5 h).

Although runnerless and near‐runnerless phenotypes have long been known in so‐called “ever‐bearing” cultivars of octoploid strawberry (Darrow, 1929, 1966), the runnerless phenotype of 55C032P001 and other perpetual flowering descendants of the Wasatch ecotype have apparently never been documented (Figures 1, 2, 3). The emergence of that transgressive phenotype among Shasta × Wasatch progeny must have been unanticipated because both parents are prolific runner producers (Bringhurst and Voth, 1980). The mean RS for Shasta was 3.0 in our study (Table 1; Supporting Information S1). The original Wasatch ecotype disappeared from the UC collection long ago; however, the ecotype recollected by J. Hancock et al. (2001) from the original Wasatch population (BT3; PI612491) was found to have the prolific and unrestrained runnering phenotype typical of *F. virginiana* spp. *glauca*. The mean RS for BT3 was 4.83 in our study (Supporting Information S1).

### 3.3 Physical mapping of the *PF* locus uncovered the persistence of linkage‐dragged DNA of the wild donor

**FIGURE 5:**
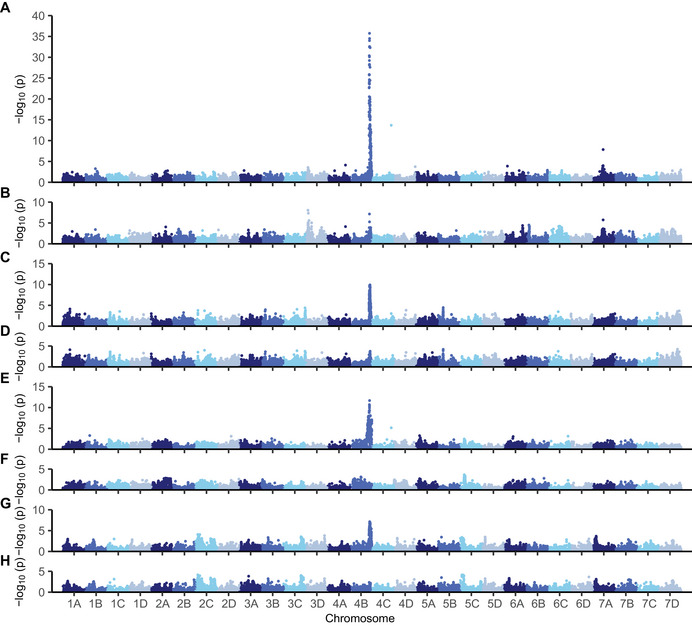
Manhattan plots displaying Axiom 50,000 array‐genotyped SNPs associated with flowering or runnering phenotypes across the octoploid strawberry genome. There are four pairs of plots (A–B, C–D, E–F, and G–H) displaying statistics for genome‐wide association study (GWAS) without fixed effects (upper panel in each pair) and where the *PF*‐associated single nucleotide polymorphism (SNP) AX‐184947290 was incorporated as a fixed effect (lower panel in each pair). (A–B) GWAS for flowering habit among 932 strawberry diversity panel individuals. (C–D) GWAS for runner score among 932 strawberry diversity panel individuals. (E–F) GWAS for runner count among 183 17C321P015 × UCD Royal Royce F_2_ and 87 17C321P015 × 55C032P001 F_2_ progeny. (G–H) GWAS for runner count among 372 full‐sib progeny developed from crosses between elite UC parents.

**FIGURE 6:**
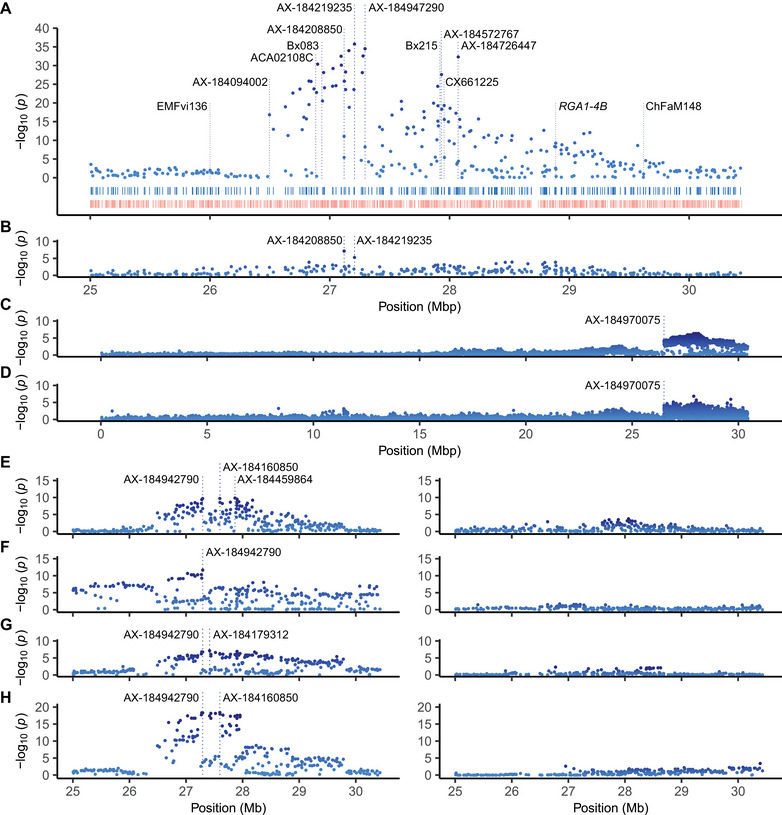
Manhattan plots displaying Axiom 50,000 or 850,000 array‐genotyped single nucleotide polymorphisms (SNPs) associated with flowering or runnering phenotypes on chromosome 4B. The physical locations of several array‐genotyped SNPs (identified by AX prefixes), previously genetically mapped DNA markers (identified by other labels), and *RGA1‐4B* (a *REPRESSOR OF GA1* homoeolog) are shown in the UCD Royal Royce genome. The Manhattan plots shown in (B) and the right hand column of (E–H) display associations identified when AX‐184947290 was used as a fixed effect. (A) SNPs associated with flowering habit observed among 932 strawberry diversity panel (SDP) individuals. The upper rug plot shows the physical positions of 322 Axiom 50,000 array SNPs, whereas the lower rug plot shows the positions of 905 annotated genes spanning Mb 25.0–30.4 in the UCD Royal Royce genome. (B) SNPs associated with flowering habit among 932 SDP individuals identified by GWAS using AX‐184947290 as a fixed effect. (C) SNPs associated with flowering habit among 66 UC individuals genotyped with the 850,000 array. (D) SNPs associated with flowering habit among 194 SDP individuals genotyped with the 850,000 array. (E) SNPs associated with runner score among 932 SDP individuals. (F) SNPs associated with runner count among 183 17C321P015 × UCD Royal Royce F_2_ and 87 17C321P015 × 55C032P001 F_2_ progeny. (G) SNPs associated with runner count among 372 full‐sib progeny developed from crosses between elite UC parents. (H) SNPs associated with inflorescence count among 372 full‐sib progeny developed from crosses between elite UC parents.

We physically mapped the *PF* locus to the lower arm of chromosome 4B using associations between octoploid genome‐anchored 50,000 array SNPs and long‐day flowering habit classifications of SDP individuals (Figures 5A and 6A). Statistically significant associations were not observed elsewhere in the genome apart from singletons on chromosomes 4C and 7A that were identified in our initial genome‐wide search and subsequently found to be false‐positives (Figure 5A). The lead *PF*‐associated SNPs were AX‐184219235 (−log10(p) = 35.7; bp 27,206,278) and AX‐184947290 (−log10(p) = 34.5; bp 27,294,311).

The pattern of LD decay for *PF*‐associated SNPs was mostly smooth in the SDP population (Figure 6A). LD steadily decayed upstream of AX‐184947290 to an LD cliff (AX‐184094002; bp 26,496,157) and downstream of AX‐184947290 to the end of the chromosome. There was a trough and irregular pattern of LD decay immediately downstream of AX‐184947290 (Figure 6A). Two SNPs downstream of the trough were strongly associated with the *PF* locus, AX‐184572767 (−log10(p) = 27.69; bp 27,930,755) and AX‐184726447 (−log10(p) = 32.28; bp 28,070,877). Those associations were likely caused by the highly admixed structure of the SDP population (Hou et al., 2021), not by the segregation of a second locus, as shown below.

Because the effect of the *PF* locus dwarfs could mask the effects of other loci affecting photoperiod‐dependent flowering, GWAS was repeated using *PF*‐associated SNPs as fixed effects, specifically AX‐184219235 or AX‐184947290 alone; AX‐184219235 and AX‐184947290 in combination; and AX‐184219235, AX‐184947290, and AX‐184208850 in combination (Figures 5B and 6B). These were the three most highly predictive *PF*‐associated SNPs for flowering habit in the SDP. When AX‐184947290 alone was used as a fixed effect for GWAS, the effects of SNP singletons on chromosomes 4C and 7A, the SNP outliers downstream of AX‐184947290 on chromosome 4B (AX‐184572767 and AX‐184726447), and other *PF*‐associated SNPs on chromosome 4B either nearly or completely disappeared (Figures 5B and 6B). The effects of AX‐184208850 and AX‐184219235 were slightly above the genetic background when AX‐184947290 was used as a fixed effect and vice versa for the others (Figure 6B). Our results suggest that the fixed effects captured haplotypic diversity in the highly admixed SDP population (Hou et al., 2021) and that the pattern of LD decay observed in the SDP population was caused by the segregation of a single gene in strong LD with AX‐184947290 (bp 27,294,311).

We genotyped a small sample of SD and DN UC individuals (n=66) and a slightly larger sample of more diverse SD and DN individuals (n=194) with an 850,000 SNP array to graphically genotype the Wasatch introgression on chromosome 4B (Figure 6C). While GWAS analyses of those small samples were underpowered and insufficient for accurately pinpointing the physical location of *PF*, the 850,000 array saturated the Wasatch introgression with 2600 SNPs, clearly identified a stable recombination breakpoint upstream of the *PF* locus (the upper boundary of the Wasatch introgression), and showed that Wasatch DNA has persisted and survived selection for perpetual flowering from the recombination breakpoint to the end of the chromosome since the original hybrid (Shasta × Wasatch) was developed in 1955 (Figure 6C). The upper boundary of the Wasatch introgression was marked by an LD cliff where significance dropped to near zero immediately upstream of the 850,000 array SNP AX‐184970075 (bp 26,490,375) (Figure 6A,C,D). The recombination breakpoint was predicted to reside between AX‐184284360 (bp 26,484,790) and AX‐184970075 (26,490,375) among individuals genotyped with the 850,000 SNP array. That interval was inside the interval (bp 26,480,809–26,496,157) where the recombination breakpoint was predicted to reside in individuals genotyped with the 50,000 SNP array.

The stability of the upper boundary suggests that the location of crossovers has been highly constrained in Wasatch descendants that inherited the *PF* allele (Figure 6A,C,D). We observed runs‐of‐homozygosity and suppressed recombination (a recombination cold spot) immediately upstream of the recombination breakpoint (Figure 6A,C,D). The LD pattern upstream of the recombination breakpoint could correlate with structural variation between SD recipients and the Wasatch donor, pedigree inbreeding caused by backcrossing to closely related SD recipients, or a combination thereof (Ceballos et al., 2018; Wright & Andolfatto, 2008).

### 3.4 SNP haplotypes shed light on the *PF*‐associated LD block found in Wastach descendants

**FIGURE 7:**
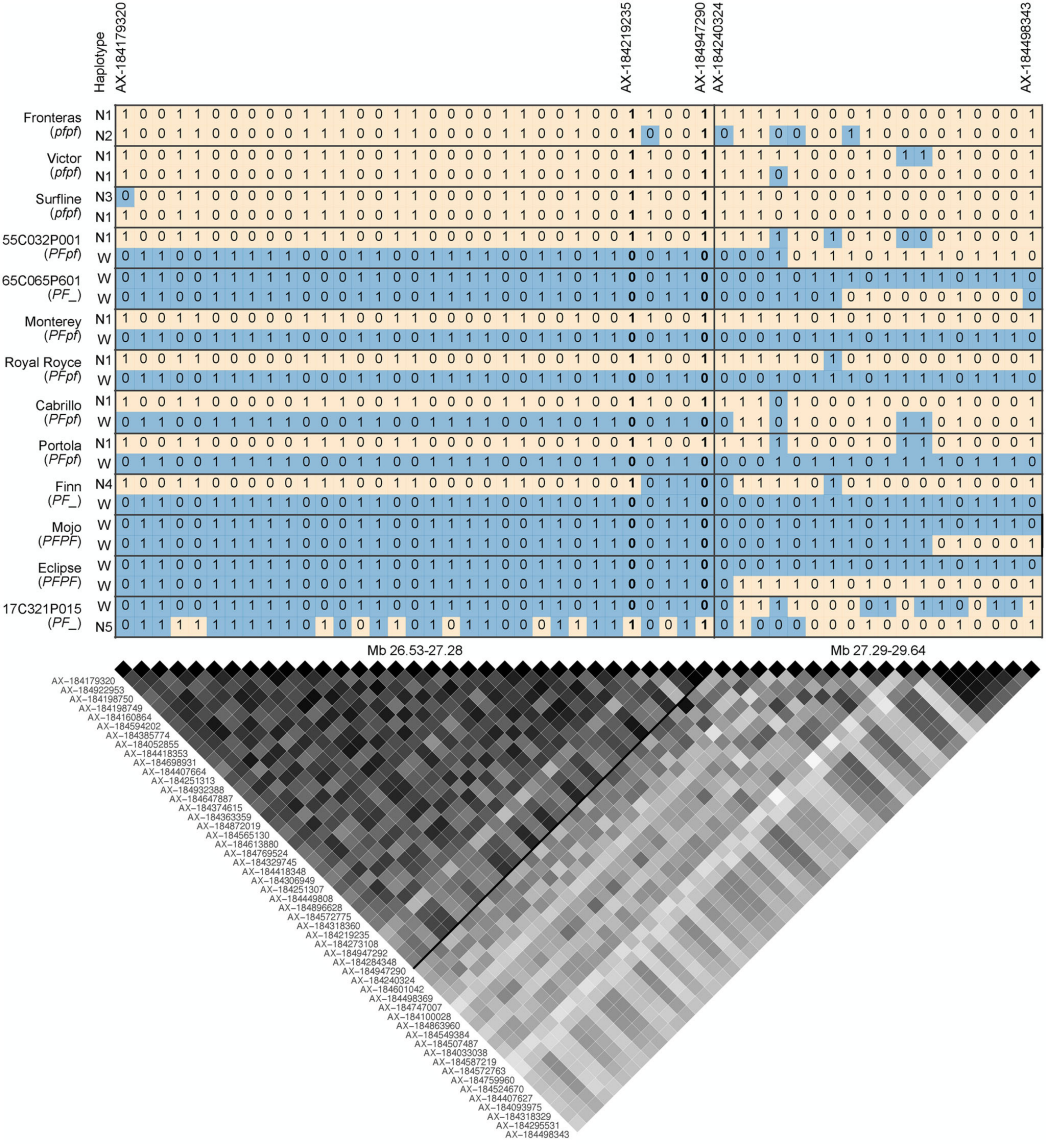
Haplotypes for 51 single nucleotide polymorphisms (SNPs) flanking the *PF* locus on chromosome 4B (Mb 26.53–29.64) among 808 strawberry diversity panel (SDP) individuals phenotyped for long day flowering habit. The selected SNPs had minor allele frequencies ≥0.3 and exceeded the false‐discovery‐rate (FDR)‐corrected significance threshold of 2 for runner score genome‐wide association study (GWAS) in the SDP population ($-\log_{10}$ *p*‐values ≥ 2). The heatmap displays squared correlation coefficient ($R^{2}$) linkage disequilibrium (LD) statistics among the 51 SNPs, where $R^{2}=1$ when LD is perfect (black cells), $R^{2}=0$ when LD is absent (white cells), and cell darkness increases as $R^{2}$ increases. The 51‐SNP LD block was split into a 33‐SNP LD block upstream of AX‐184240324 and an 18‐SNP LD block downstream of AX‐ 184947290 (identified by a solid line between the upper and lower LD blocks). Haplotypes are shown for individuals carrying six out of 400 unique haplotypes in the 33‐SNP LD block, including the Wasatch haplotype (W), a common short‐day haplotype (N1), and four others (N2–N5). *PF* genotypes are shown in parentheses below the names of each individual. Axiom 50,000 array SNP marker names are displayed in the upper panel for a select subset of the 51 SNPs.

The SDP population included 423 DN individuals developed at UCD between 1955 and 2021 (Wasatch descendants) and 396 SD individuals developed at UCD between 1935 and 2021 (Figure 4; Supporting Information S1). With 66 generations of historical recombination (breeding), hybrid offspring of 563 unique pedigrees (FS crosses) among 498 parents in the SDP and FS populations combined, and highly accurate flowering habit classifications of SDP individuals, we anticipated that GWAS might pinpoint the physical position of the gene encoded by *PF*; however, recombination breakpoints were scarce from the upper boundary of Wasatch introgression (Mb 26.53) to the lower boundary (Mb 27.28–27.29) of the LD block predicted to harbor *PF* (Figure 7; Supporting Information S6).

To assess the strength and pattern of LD and search for recombination breakpoints, haplotypes of 51 *PF*‐associated SNPs were phased among 808 SDP individuals (Figure 7; Supporting Information S6). The SNPs selected for this analysis had minor allele frequencies (MAFs) greater than 0.3 and GWAS effects for RS that exceeded a false‐discovery‐rate (FDR) significance threshold of $-\log_{10}\left(\text{p-value}\right)=2$ among SDP population individuals (Figure 6C, D). We identified 400 unique 51‐SNP haplotypes among 808 SDP individuals (1616 haplotypes; Supporting Information S6). The five most common haplotypes were observed 74 to 234 times each and accounted for 700 of the 1616 haplotypes (43.3%), whereas 291 haplotypes were only observed once. The 51‐SNP Wasatch haplotype was observed in 87% of the individuals classified as DN (*PF*_).

**FIGURE 8:**
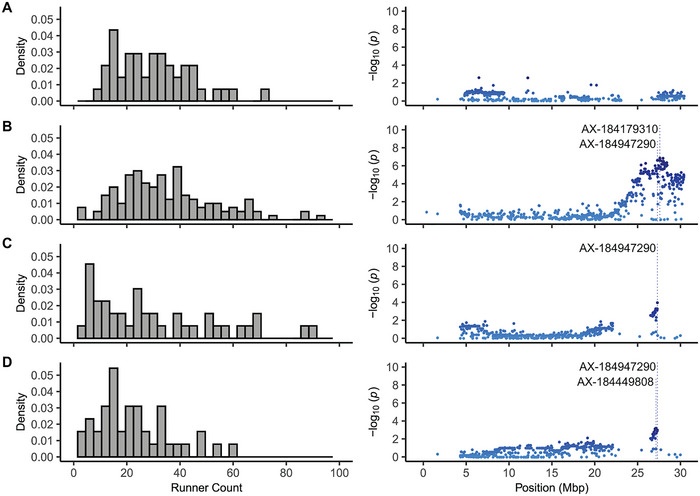
Variation for runner count (left column) and Manhattan plots displaying Axiom 50,000 array‐genotyped single nucleotide polymorphisms (SNPs) associated with runner count on chromosome 4B (right column) in four F_2_ populations. (A) 17C321P015 × UCD Royal Royce F_2_ (21S950 originating by self‐pollinating the 19A907P022 F_1_). (B) 17C321P015 × UCD Royal Royce F_2_ (21S951 originated by self‐pollinating the 19A907P024 F_1_). (C) 17C321P015 × 55C032P001 F_2_ (21S952 originated by self‐pollinating the 19A908P012 F_1_). (D) 17C321P015 × 55C032P001 F_2_ (21S953 originated by self‐pollinating the 19A908P053 F_1_). The lead SNPs are labeled.

Strong LD was observed among the 33 phased SNPs upstream of AX‐184240324 (Mb 27.29; Figure 7). LD was markedly weaker among the 18 phased SNPs downstream of AX‐184947290 (Mb 27.28). We identified 182 unique 33‐SNP haplotypes, including a common Wasatch haplotype (W) in the upper LD block (Figure 7). The boundary between the upper and lower LD blocks was supported by the absence of *PF* segregation among 17C321P015 × 55C032P001 F_2_ progeny and among FS progeny developed from crosses between UC Eclipse and SD cultivars (Figure 8; Supporting Information S7). UC Eclipse carries a recombination breakpoint immediately downstream of AX‐184240324; hence, we concluded that *PF* is found upstream of the upper boundary of the lower LD block. Cabrillo and UCD Finn share that recombination breakpoint (Supporting Information S7). When combined with insights gained from unique recombination breakpoints among F_2_ progeny, SNP haplotypes of SDP individuals, and the presence or absence of *PF* segregation among F_2_ progeny and UC Eclipse × SD FS progeny, we concluded that the *PF* gene must be found between AX‐184021395 (Mb 26.40) and AX‐184240324 (Mb 27.28) on chromosome 4B (Figures 6, 7, 8; Supporting Information S6–S8).

Our search of annotations in UCD Royal Royce genome assemblies (both haplotypes) identified 193 genes spanning bp 26,401,121–27,418,663 on chromosome 4B (Supporting Information S9). That search did not uncover any genes known or predicted to regulate flowering in angiosperms (Hempel et al., 1997). We did, however, discover a homolog of *REPRESSOR OF GA1* on chromosome 4B (*RGA1‐4B*) downstream of *PF*. *RGA1‐4B* gene identifiers in the haplotype‐phased UCD Royal Royce genome assemblies are Fxa4Bg203107 (haplotype 2; Mb 28.83–28.94) and Fxa4Bg103085 (haplotype 1; Mb 29.15–29.25). Homoeologs were also identified in the other three genomes (Figure S2). Studies in *F. vesca* identified a *REPRESSOR OF GA1* (*FveRGA1*) gene on chromosome 4 as a causal determinant of the runnerless phenotype of Yellow Wonder (Caruana et al., 2018; Tenreira et al., 2017). Hawaii 4 develops runners under long days, whereas Yellow Wonder does not.

### 3.5 Genome‐wide association studies identified a single QTL for runnering

As anticipated, we observed LD blocks of *PF*‐coincident RS and count‐associated SNPs on the distal end of chromosome 4B in GWASs of the SDP, F_2_, and FS populations (Figures 5C–H and 6E–G). Those *PF*‐associated SNPs identify the *PFRU* QTL previously identified by genetic mapping (Gaston et al., 2013). Significant associations with RS or count QTL were not observed elsewhere in the genome (Figure 5). That was unanticipated, especially in the SDP and F_2_ populations. The absence of QTL was less surprising in the FS population because the parents were elite individuals with runnering phenotypes that are less extreme than the exotic parents of the F_2_ populations and exotic individuals in the SDP population (Figure 4; Table 1).

Our initial hypothesis was that the runnerless phenotype of 55C032P001 might be caused by a single gene mutation independent of the Wasatch *PF* mutation. This was based on the finding in woodland strawberry (*F. vesca*) that the runnerless phenotype was caused by mutations in a single gene (*R*) independent of the gene (*S*) that controls the perpetual flowering phenotype (Brown & Wareing, 1965). We did not, however, uncover an independent mutation for the runnerless phenotype in our GWASs of 17C321P015 × 55C032P001 F_2_ progeny developed by self‐pollinating runnerless F_1_ individuals (Figures 1 and 5; Table 1). Our analyses solely pointed to the *PFRU* QTL (Gaston et al., 2013) and showed that the genetic architecture of runnering is complex in octoploid strawberry (Figure 8E–G; Table 3).

**TABLE 3:** Statistics^a^ for an A/G single nucleotide polymorphisms (SNP) marker (AX‐184947290) associated with the *PF* locus on chromosome 4B.

Population^b^	Trait^c^	${\bar{y}}_{\text{GG}}$	${\bar{y}}_{\text{AG}}$	${\bar{y}}_{\text{AA}}$	$\hat{a}$	$\hat{d}$	$|\hat{d}/\hat{a}|$	PVE (%)
Diversity panel	Runner score	3.7	3.1	3.0	0.4	−0.2	0.51	14.9
Pooled F_2_	Runner count	31.5	20.8	15.0	8.3	−2.4	0.29	29.1
21S950 F_2_	Runner count	—	—	16.0	—	—	—	—
21S951 F_2_	Runner count	38.6	27.9	20.5	9.1	−1.7	0.19	24.6
21S952 F_2_	Runner count	27.7	14.2	9.9	8.9	−4.6	0.51	31.1
21S953 F_2_	Runner count	22.1	13.7	10.8	5.6	−2.8	0.49	22.6
Full‐sib families	Runner score	3.6	3.2	2.8	0.3	0.0	0.06	7.1
	Runner count	50.7	37.6	28.5	11.1	−2.0	0.18	15.0
	Inflorescence count	0.0	4.5	10.6	−5.3	−0.8	0.14	46.7

^a^ ${\bar{y}}_{\text{GG}}$, ${\bar{y}}_{\text{AG}}$, and ${\bar{y}}_{\text{AA}}$ are estimates of SNP marker genotypic means. $\hat{a}=\left({\bar{y}}_{\text{GG}}-{\bar{y}}_{\text{AA}}\right)/2$ is the additive effect, $\hat{d}={\bar{y}}_{\text{AG}}-\left({\bar{y}}_{\text{GG}}+{\bar{y}}_{\text{AA}}\right)/2$ is the dominance effect, and $|\hat{d}/\hat{a}|$ is the degree‐of‐dominance of the AX‐184947290 SNP marker locus. PVE is an estimate of the phenotypic variances explained by the SNP marker locus.

^b^ Statistics were estimated from clonal replicates of strawberry diversity panel individuals (n=932), unreplicated seed‐propagated 17C321P015 × Royal Royce (21S950 and 21S951) and 17C321P015 × 55C032P001 (21S952 and 21S953) F_2_ progeny (n=270), and unreplicated seed‐propagated full‐sib progeny (n=372) developed from nine crosses between elite parents with contrasting runnering phenotypes (Table 1).

^b^ The flowering habits of SDP individuals were classified as short‐day or day‐neutral from the absence or presence of flowering, respectively, under long daylengths (13.5–14.5 h) in Winters, CA. The runner scores of strawberry diversity panel (SDP) individuals were recorded over a period of 6 years in Winters, CA using an ordinal scale, where 1 = *runnerless*, 2 = *weak runnering*, 3 = *intermediate runnering*, 4 = *strong runnering*, and 5 = *extreme runnering* (see Figure 3A). F_2_ and full‐sib progeny were phenotyped by counting the number of primary runners observed in Davis or Winters, CA, respectively, within 1 week of the summer solstice (the daylength was 14.5 h).

**FIGURE 9:**
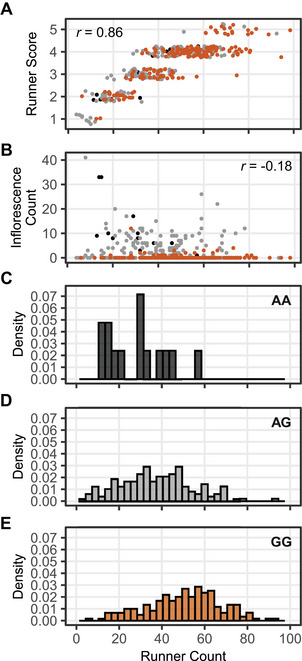
Variation for runner score, runner count, and inflorescence count among 372 full‐sib progeny from nine families phenotyped July 1, 2024 in Davis, CA (14.5 h photoperiod) from seedlings transplanted on October 15, 2023. *PF* and the *PF*‐associated single nucleotide polymorphism (SNP) AX‐18497290 segregated in this population. The parents were elite UC short‐day and day‐neutral individuals (Table 1). (A–E) AX‐184947290 SNP genotypes are color coded dark gray (AA), light gray (AG), and orange (GG). (A) The runner count by runner score distribution. (B) The runner count by inflorescence count distribution. (C–E) The runner count distributions among AA, AG, and GG individuals.

The statistics reported throughout this paper for runner count in the F_2_ and FS populations were estimated from phenotypes observed at the summer solstice when the photoperiod was 14.5 h (Figures 5, 6, 8, and 9). We selected that time point because the effect of *PF* locus on runnering was greatest at the summer solstice (Figure S1). The temporal effect of the *PF* locus is shown in Manhattan plots developed for GWAS analyses of phenotypes observed among F_2_ progeny on seven dates, from March 26 (12.3 h photoperiod) to July 6, 2022 (14.5 h photoperiod) (Figure S1). As anticipated, the pleiotropic effect of the *PF* locus on runnering was weakest and nonsignificant early in the season (March 26 –May 6) and steadily increased through the summer solstice (June 21).

The patterns of LD decay for RS or count‐associated SNPs on chromosome 4B differed in the SDP, F_2_, and FS populations because of differences in population structure, pedigree inbreeding, recombination, and selection history (Figure 6E–G; Supporting Information S6–S8). They provided different pieces to the puzzle for understanding the pleiotropic effects of the *PF* locus on runnering in elite and exotic genetic backgrounds (Table 3). The patterns of LD decay for flowering‐habit‐ and RS‐associated SNPs on chromosome 4B were virtually identical in the highly admixed SDP population (Figure 4 Supporting Information S6). The lead SNP AX‐184947290 was predicted to be closest to the gene encoded by *PF* (Figure 6E). Using that SNP as a predictor, the *PF* locus was estimated to explain approximately 22.1% of the genetic variance and 14.9% of the phenotypic variance for RS in the SDP population (Table 3). As observed for flowering habit, when AX‐184947290 was used as a fixed effect, the effects of other *PF*‐associated SNPs disappeared (Figures 5D and 6E).

The strongest GWAS signals and smoothest patterns of LD decay for runnering were observed in the F_2_ populations (Figures 5, 6, and 8A–D). Figures 5E and 6F display Manhattan plots for analyses of the F_2_ populations combined. Figure 8 displays the runner count phenotypic distributions and chromosome 4B Manhattan plots for analyses of individual F_2_ populations. The combined F_2_ population analysis produced sharper and cleaner GWAS signals than those observed in the SDP or FS populations because the phenotypes of the parents were extreme, haplotypic variation was minimum, and the parents carried unique recombination breakpoints (Table 1; Supporting Information S7 and S8). We observed an LD cliff immediately downstream of AX‐184942790 in combined F_2_ and individual 17C321P015 × 55C032P001 F_2_ analyses with comparatively flat LD beyond the LD cliff to the end of the chromosome, in contrast to the choppy pattern of LD decay observed immediately downstream of AX‐184942790 in the SDP population (Figures 6E,‐F and 8B–D).

We discovered that individuals in the 21S950 (17C321P015 × UCD Royal Royce) F_2_ family were homozygous for Wasatch alleles from the upper boundary of the Wasatch introgression (AX‐184251339; bp 25,006,055) to AX‐184418344 (bp 27,417,624), the 50,000 array SNP immediately downstream of AX‐184947290 (Supporting Information S8). There was an absence of runner‐count associated SNPs across the Mb 25.0–30.4 genomic segment in that F_2_ family; hence, we concluded that the gene was likely located upstream of bp 27,417,624 (Figure 8A). By contrast, significant runner count‐associated SNPs were observed across the entire genomic segment (Mb 25.0–30.3) in the other 17C321P015 × UCD Royal Royce F_2_ family (21S951). High‐density graphical genotypes and haplotypes showed that AX‐184947290 and numerous other SNPs upstream of AX‐184418344 (bp 27,417,624) segregated in that F_2_ family (Supporting Information S7 and S8). The LD blocks transmitted in those F_2_ individuals were wide, thereby creating a comparatively flat pattern of LD decay across the entire genomic segment in the 21S951 F_2_ family (Figure 8B).

The runnerless 17C321P015 × 55C032P001 F_1_ individuals that we selected and self‐pollinated (19A908P012 and 19A908P053) were discovered to be homozygous upstream of AX‐184198749 (bp 26,691,998) and downstream of AX‐184947290 (bp 27,294,311) (Supporting Information S7 and S8), and that genomic segment segregated in both of those F_2_ families (21S952 and 21S953; Table 1). Fourteen SNPs in the segregating genomic segment were significantly associated with runner count (Figure 8C,D). Hence, when combined with our association studies in the 17C321P015 × UCD Royal Royce F_2_ families, we concluded that the gene encoded by *PF* must be located upstream of AX‐184418344 (bp 27,417,624) and proximal to AX‐184947290 (Supporting Information S7 and S8).

Although the dominant Wasatch *PF* allele partially suppresses runnering under long days, we found that *PF*_ individuals can have dramatically different runnering phenotypes and that runnering is not always suppressed in *PF*_ individuals, for example, the runnering phenotype of 21C221P054 (*PFpf*) was extreme (RS = 5.0) and virtually identical to that of the wild parent (Figures 2, 3, 8, and 9). At the other extreme, 20C420P006 (*PFpf*) was runnerless (RS = 1.0; Figure 2). Our analyses of individual F_2_ populations showed that the phenotypic variation observed for runnering among SD (*pfpf*) and DN (*PF*_) individuals cannot be explained by the segregation of *PF* alone (Figures 2, 3, and 8; Table 3). The runner count distributions were strikingly similar among the four F_2_ families; however, phenotypes ranged from runnerless to prolific in three of the four (21S951, 21S952, and 21S953) and weak (10 runners/plant was the minimum) to prolific in the other (21S950) (Figure 8). GWAS for runner count showed that the 21S950 F_2_ family did not segregate for the *PF* gene, whereas the other three F_2_ families did (Figure 8; Table 3; Supporting Information S7 and S8).

The FS population combined progeny from nine crosses between elite parents with runnering phenotypes typical of commercially important cultivars (RSs in the 2.5–4.0 range; Table 1; Figure 9). The individuals in those families were phenotyped for runner and inflorescence count. Association studies of the latter shed light on the pleiotropic effect of the *PF* locus on runner count in elite genetic backgrounds (Figure 6H). Significant inflorescence count–associated SNPs identify the boundaries of the *PF*‐associated LD blocks transmitted by the parents. The pattern of LD decay for flowering habit was comparatively flat from Mb 26.5 to 28.0 among FSs because of limited recombination. We observed a distinct LD cliff upstream of *PF* aligned with the stable recombination coldspot found in Wasatch descendants (Figure 6H). That pattern clearly shows that LD blocks spanning at least a 0.5 Mb upstream and downstream of the *PF* locus (Mb 27.3) are typically transmitted in segregating populations, partly as a function of limited recombination and partly as a function of shared ancestry (pedigree inbreeding) among elite parents (Table 1) As anticipated, the effect of the *PF* locus was approximately three‐fold greater for inflorescence than runner count (Figure 6G,H; Table 3). Consequently, the LD decay for runner count was comparatively flat and lacked LD cliffs in the FS population (Figure 6G).

We found that RS was a directionally accurate predictor of runner count, particularly among individuals in the lower tails of both distributions (Figure 9). The runner counts of seed‐propagated FS progeny observed near the summer solstice (14.5 photoperiod) were highly correlated with RS (r=0.86). Notably, the runner count ranges were narrowest among FS progeny with RSs in the runnerless to near‐runnerless range (1–2). The runnering phenotypes of at least 18.6% of the FS progeny were transgressive (1 ≤ RS ≤ 2). Using the *PF*‐associated SNP AX‐184947290 as a predictor, of 58 individuals with RSs = 2, seven were *PFPF*, 29 were *PFpf*, and 22 were *pfpf*; hence, reduced runnering phenotypes were recovered among both SD and DN individuals.

### 3.6 The pleiotropic effect of the *PERPETUAL FLOWERING* locus on runnering across a broad cross‐section of octoploid genetic resources

Our data support the hypothesis that the *PFRU* QTL for runnering previously identified by Gaston et al. (2013) was likely caused by the pleitropic effect of a single gene (*PF*). Using the lead SNP identified by GWAS (AX‐184947290), we estimated that the *PF* locus explained 7.1%–31.1% of the phenotypic variation for RS or count (Table 3). The Wasatch allele has a dominant (Mendelian) effect on long‐day flowering habit; however, the pleiotropic effects of the *PF* allele on runnering are quantitative and incompletely dominant: $|\hat{d}/\hat{a}|$ ranged from 0.18 in the elite × elite FS population to 0.51 in the SDP population (Table 3).

The *PF* locus is well‐known to affect the timing of the photoperiod‐dependent inhibition of flowering and discrete transition from sexual to asexual reproduction in SD (*pfpf*) plants (Ahmadi et al., 1990; Gaston et al., 2013; Sønsteby and Heide, 2006). While neither are observed in DN (*PF*_) plants (Gaston et al., 2013; Sønsteby and Heide, 2006), both SD and DN plants runner under a wide range of conditions, including short and long days, and when producing fruit, which is why runner trimming is commonly practiced and necessary to promote flowering and maximize yield in fruit production (Figures 1, 2, 3). We observed the complete range of phenotypes, from runnerless (RS = 1.0) to extreme runnering (RS = 5.0), among the diverse sample of SD and DN individuals assembled for the SDP and parents of F_2_ families segregating for *PF* alleles and runnering phenotypes (Table 1; Figure 3; Supporting Information S1).

The runnering phenotypes of most of the wild species ecotypes in our study were aggressive (RS > 4.0), as exemplified by Frederik 9, Hinesburg, Harris Spring, and Yaquina A in Figure 1 (Supporting Information S1). The median RS was $\tilde{y}=4.8$ among *F. chiloensis* and $\tilde{y}=4.8$ among *F. virginiana* ecotypes. The RS means for 33 of the 38 octoploid ecotypes phenotyped in our study were in the 4.0–5.0 range, and only one ($\bar{y}=3.0$; Scott's Creek) was less than the SDP population mean ($\bar{y}=3.38$).

The RS distribution was negatively skewed among SD and approximately normal among DN individuals in the SDP population (Figure 3). The RS mean was significantly greater for SD (*pfpf*) individuals (${\bar{y}}_{SD}=3.68$) than DN (*PF*_) individuals (${\bar{y}}_{\text{DN}}=3.07$; p≤0.0001). Similarly, the RS median was greater for SD individuals (${\tilde{y}}_{\text{SD}}=3.95$) than DN individuals (${\tilde{y}}_{\text{DN}}=2.97$) (Figure 3; Supporting Information S1). These differences broadly reflect the pleiotropic effect of the *PF* locus on runnering over an entire growing season in our low‐elevation nursery production (clonal propagation) environment (Figures 1 and 2).

As shown for a broad cross‐section of octoploid genetic resources, DN (*PF_*) individuals tend to produce fewer runners than SD (*pfpf*) individuals in nursery production (Figure 3; Supporting Information S1). Of 478 SD individuals in the SDP population, 97.5% had RSs in the 2.5–5.0 range. The 12 SD individuals in the weak runnering range (1.0–2.5) include the cultivars Elsanta (RS = 1.2), Howard 17 (RS = 2.0), Senga Sengana (RS = 2.0), and Fairfax (RS = 2.0). The other eight are UC individuals developed in 2018 that were purposefully selected for reduced runnering (Supporting Information S1).

We observed a wide range of runnering phenotypes among DN individuals known to be homozygous or heterozygous for the dominant Wasatch *PF* allele, for example, 20C420P006 (*PFpf*; RS = 1.0), UCD Moxie (*PFpf*; RS = 2.0), UC Eclipse (*PFPF*; RS = 3.4), and Monterey (*PFpf*; RS = 4.0) (Figure 2). Of 444 individuals classified as DN in the SDP population, 80.0% had runners scores in the 2.5–5.0 range (Figure 3; S1). The percentage of individuals in the weak runnering range (1.0–2.5) was greater for DN (20.0%) than SD (2.5%) individuals. Weak runnering DN cultivars were uncommon (the runnering phenotypes of 30 of the 40 DN cultivars screened in our study were intermediate to strong).

DN cultivars carrying the Wasatch *PF* allele have occasionally been described as poor runner producers, but cannot be broadly classified as poor runnering, for example, Hossain et al. (2019) described Albion (*PFpf*) as a “poor runner producer” and depicted a runnerless Albion plant. Our data do not support that characterization. Albion produced an above average number of runners (RS = 3.3), runners in fruit and bare‐root plant production environments, and has been clonally propagated on a large‐scale for commercial production since 1997 (Figure 2; Supporting Information S1). Our data show that the runnering phenotypes of DN (*PF*_) individuals temporally vary and span the entire range, from runnerless to extreme (Figures 3, 8, and 9).

### 3.7 Accuracy of genomic selection for runnering

The *PF* locus explained 22.1% of the genetic variance for runnering in the SDP population and was found to be necessary but insufficient for predicting runnering phenotypes in strawberry populations segregating for dominant and recessive *PF* alleles (Table 3; Figures 1 and 2). With as much as 78% of the heritability for runnering missing (unexplained by segregation of the *PF* locus), we explored the merit of applying genomic selection for runnering by testing different combinations of training and validation populations with and without correcting for the fixed effect of the *PF* locus (Table 4). We specifically sought to identify how and where genomic selection for runnering might be cost effectively applied in a strawberry breeding pipeline to identify runnerless individuals as selection candidates for seed‐propagated cultivars and near‐runnerless individuals as selection candidates for clone‐propagated cultivars. The strategy for the latter is to identify individuals near the lower limit of commercially viable clonal propagation, specifically those with RSs in the 2.0–2.5 range (Figures 3 and 9).

**TABLE 4:** Genomic predictive ability ($r_{\hat{a},\bar{y}}$) and prediction accuracy ($r_{\hat{a},\bar{y}}/h$) for runner score or count within and between strawberry populations estimated by cross‐validation^a^ with and without correcting for the effect of the *PF* locus^b^.

Population^c^		PF Uncorrected		PF Corrected	
Training	Validation	$r_{\hat{a},\bar{y}}$	$r_{\hat{a},\bar{y}}/h$	$r_{\hat{a},\bar{y}}$	$r_{\hat{a},\bar{y}}/h$
SDP	SDP	0.59	0.76	0.60	0.77
SDP‐UC	SDP‐UC	0.47	0.62	0.50	0.66
FS	FS	0.55	0.75	0.58	0.79
SDP	FS	0.33	0.45	0.43	0.60
SDP‐UC	FS	0.25	0.34	0.39	0.53

^a^ Cross‐validation was done by randomly drawing 80% of the individuals within a training population to predict genomic‐estimated breeding values ($\hat{a}$) of validation population individuals. The prediction accuracy was estimated by $r_{\hat{a},\bar{y}}/h$, where $r_{\hat{a},\bar{y}}$ is the simple correlation between the observed phenotype (y) and $\hat{a}$, $h=\sqrt{h^{2}}$, $h^{2}$ is the genomic‐estimated narrow‐sense heritability, y was the either the runner count for unreplicated seed‐propagated full‐sib (FS) individuals or the estimated marginal mean for runner score for clonally propagated strawberry diversity panel (SDP) individuals over years. Cross‐validation statistics were estimated from 1000 randomly drawn samples of training population individuals with replacement. When predicting within a population (e.g., within the SDP population), $\hat{a}$ was estimated for individuals that were not sampled in each iteration (20% of the individuals within the population). When predicting between populations, $\hat{a}$ was estimated for every individual in the validation population.

^b^ The *PF*‐associated single nucleotide polymorphism (SNP) AX‐184947290 was used as a fixed effect to correct for the effect of the *PF* locus on runner score or count.

^c^ The populations are the SDP (n=932), a population‐specific subset of cultivars and other elite SDP individuals developed at the University of California (UC), Davis (SDP‐UC; n=672), 17C321P015 × Royal Royce and 17C321P015 × 55C032P001 F_2_ families (n=270), and nine FS families (n=372) developed from crosses between elite UC parents.

Because the SDP and FS family training populations segregated for dominant and recessive *PF* alleles, we used a *PF*‐associated SNP marker (AX‐184947290) to correct for the fixed effect of the *PF* locus (Tables 1 and 4). The inclusion of AX‐184947290 as a fixed effect increased the accuracy of genomic prediction across the training and validation population combinations tested (Table 4). The accuracy increase gained by *PF*‐correction was greater for the between population combinations (training → validation = SDP → FS and SDP‐UC → FS) than the within population combinations tested in our study, for example, SDP → SDP (Table 4). As anticipated, directly correcting for the fixed effect of the *PF* locus is necessary for maximizing genomic prediction accuracy when selecting for runnering in populations segregating for dominant and recessive *PF* alleles (Table 4).

The clonal genetic resources associated with strawberry breeding programs typically comprise permanent to semipermanent individuals, both exotic and elite, and a continually evolving sample of selected individuals spanning several generations of within FS family selection. The 932 individuals selected for the SDP originated from 554 unique pedigrees (FS families) among 489 parents (Supporting Information S1). The phenotypic data underlying breeding program‐associated clonal genetic resources are continually updated, thereby creating ever‐increasing samples of training population data to inform parent and genomic selection (Supporting Information S1). We used the SDP to assess whether RS observations accumulated year‐over‐year among genetically diverse individuals could be used to accurately estimate breeding values for runner count among seed‐propagated elite × elite FS progeny (selection candidates before clonal propagation). We discovered that breeding values for RS were predicted with outstanding accuracy within the SDP population per se (the *PF*‐corrected estimate was $r_{\hat{a},\bar{y}}/h=0.77$). The prediction accuracy was lower when the SDP was used as the training population for predicting breeding values among elite × elite FS progeny (SDP → FS; Table 4). We note here that physiological and methodological differences could partly account for the slightly lower SDP to FS prediction accuracy. SDP individuals were clonally propagated and phenotyped in late summer using an ordinal scale, whereas FS individuals were seed propagated and phenotyped at the summer solstice for runner count.

To further explore the approach of using phenotypes of genetic resources (e.g., the SDP population) for genomic prediction of runnering breeding values in elite segregating populations, we tested a training population comprising 776 SDP individuals with UC pedigrees starting from 1969 onward (SDP‐UC), the year of origin of the first Wasatch‐derived DN cultivars, specifically Hecker, Aptos, and Brighton (Bringhurst and Voth, 1980). The SDP‐UC subset was created by eliminating 156 pre‐1969 UC, non‐UC, and other exotic individuals from the SDP (File S1). Because accuracy depends on the closeness of genetic relationships and patterns of LD between training and validation populations (Habier et al., 2007; Hickey et al., 2014; A. J. Lorenz & Smith, 2015), our hypothesis was that accuracy could be increased by using a UC‐specific subset of SDP training population individuals to predict the breeding values of FS progeny developed with elite UC parents. We discovered that RS breeding values were predicted with acceptable accuracy within the SDP‐UC training population per se (SDP‐UC → SDP‐UC); however, to our surprise, genomic prediction accuracy was lower within the SDP‐UC than the SDP training population, for example, $r_{\hat{a},\bar{y}}/h$ decreased from 0.77 in the SDP → SDP cross‐validation to 0.66 in the SDP‐UC → SDP‐UC cross‐validation (Table 4). This might be partly explained by decreased genetic variation in the SDP‐UC population.

We attributed the decreased accuracy of the SDP‐UC training population to the reduction of individuals from the upper and lower tails of the RS distribution and associated decreases in allelic diversity among loci underlying extreme phenotypes eliminated by artificial selection (Figure 9). While adding genetically distant individuals to a training population often decreases genomic prediction accuracy, we suspect that the effect of eliminating genetically distant individuals from the training population was offset by the loss of allelic variation needed for accurate genomic prediction (Habier et al., 2007; Hickey et al., 2014; A. J. Lorenz & Smith, 2015). Selection against runnering phenotypes at both extremes (RS < 2.0 and RS > 4.0) has narrowed genetic variation for runnering among modern cultivars, including those in the SDP‐UC subset. Our analysis suggests that augmenting the training population with extreme phenotypes can increase prediction accuracy, similar to what has been proposed in similar breeding scenarios where selection has significantly changed allele frequencies and eliminated unfavorable alleles (Brandariz & Bernardo, 2018; Knapp et al., 2024).

The prediction accuracy for runner count within the FS training population (FS → FS) was nearly identical to that observed within the SDP training population (Table 4). This suggested that phenotyping a nominal number of seed‐propagated (unreplicated) FS individuals for runner count should facilitate the accurate prediction of breeding values of unphenotyped FS individuals, particularly with continual updating of the training population (Hickey et al., 2014; Jannink et al., 2010; A. Lorenz & Nice, 2017). The approach of counting runners among a subset of individuals within FS families, although effective for building a training population and applying genomic selection, is time consuming, cost prohibitive, and unnecessary for identifying individuals with runnerless to near‐runnerless phenotypes, primarily because rapid visual phenotyping and phenotypic selection among seed‐propagated FS individuals was found to be highly effective (Figures 1 and 2).

## 4 DISCUSSION

Our studies were undertaken to assess the feasibility of identifying runnerless and reduced runnering selection candidates in segregating populations. Although the genetic mechanisms underlying variation for runnering were found to be complex (a quantitative black box), the broad‐sense heritability, stability, and reproducibility of runnering phenotypes were exceptional in our studies and showed that the natural genetic variation needed to develop runnerless or reduced runnering cultivars is prevalent and heritable in octoploid strawberry (Figures 1, 2, 3 and 9; Table 2). We recovered runnerless and reduced runnering phenotypes at moderately high frequencies and found them to be stable and reproducible despite the complex genetic architecture of runnering.

The pleiotropic effect of the *PF* locus on runnering was found to be highly variable, temporal, and incompletely dominant, even though the effect of the *PF* allele on flowering is highly stable, predictable, and dominant (Table 3; Figure S2; Ahmadi et al. 1990; Gaston et al. 2013; Hossain et al. 2019). From a practical breeding perspective, the pleiotropic effect of the *PF* locus on runnering is inconsequential because progeny are destined to be SD (*pfpf*) or DN (*PF_*). To state this another way, when selecting for runnering within SD or DN populations per se, the outcome of selection hinges on genetic variation caused by loci other than *PF* (Table 3). Those other loci underlie the quantitative variation and missing heritability for runnering uncovered in our study (Table 2).

Our physical mapping study facilitated the integration and cross‐referencing of findings from earlier genetic mapping studies of the *PF* locus (Honjo et al., 2016; Perrotte et al., 2016; Sargent et al., 2016; R. Spigler et al., 2008, R. B. Spigler et al., 2010; Saiga et al., 2022). This was previously difficult because of differences in linkage group and chromosome nomenclatures; the absence, inaccessibility, or nontransferability of earlier DNA marker information; and the absence of highly contiguous octoploid reference genomes and physically anchored DNA markers (Hardigan et al., 2020, 2021). Those difficulties were eliminated with the assembly and annotation of highly contiguous, haplotype‐phased octoploid genomes, the development of octoploid genome‐anchored 50,000 and 850,000 SNP arrays and medium‐density genotyping platforms, the discovery that a significant percentage of short DNA sequences could be unambiguously aligned to octoploid reference genomes, the development of a chromosome nomenclature aligned with the evolutionary history and organization of the four genomes, and the development of a Rosetta Stone for cross‐referencing linkage group and chromosome nomenclatures (Supporting Information S3; Tennessen et al., 2014; Hardigan et al., 2020, 2023; Session & Rokhsar, 2023).

When DNA sequences for previously genetically mapped *PF*‐linked simple sequence repeat (SSR) and amplification‐refractory mutation system (ARMS) markers were aligned to the UCD Royal Royce genome (Honjo et al., 2016; Perrotte et al., 2016; Sargent et al., 2016; R. Spigler et al., 2008, R. B. Spigler et al., 2010; Saiga et al., 2022), we discovered that they are located upstream or downstream of the physical position predicted for the *PF* locus (Figure 6A). Our analyses shed light on why fine‐scale genetic mapping of the *PF* locus has been challenging. We showed that recombination has been limited and that wild donor DNA has persisted in the LD block predicted to harbor the *PF* locus (Figure 8). The high density and even distribution of physically anchored SNPs on the 50,000 and 850,000 arrays used in our studies enabled us to saturate chromosome 4B with DNA markers and graphically genotype the wild donor genomic segment that has persisted in modern DN descendants of Wasatch (Figure 6; Supporting Information S7).

Using the DNA sequences for previously genetically mapped SSR and ARMS markers, the Rosetta Stone, and Royal Royce genome as a physical reference, we found that upward of 10 linkage group nomenclatures have been used to describe the location of DNA markers linked to *PF* on chromosome 4B (Figure 6A; Supporting Information S3). The linkage group identifiers used in those earlier genetic mapping studies are LGIVb‐f (Gaston et al., 2013; Perrotte et al., 2016), IV‐T‐1 (Castro et al., 2015; Honjo et al., 2016), LG‐4A (Verma et al., 2017), 4A (Cockerton et al., 2023), LG4‐D (Davik et al., 2015), LG4b (Sargent et al., 2016), Fvb4‐4 (Edger et al., 2019; Saiga et al., 2022), LGIV (R. B. Spigler et al., 2010), LG19 (R. Spigler et al., 2008), and possibly LG28 (Weebadde et al., 2008). Several of these linkage group nomenclatures, despite variation in acronyms and symbols, were found to have correctly identified chromosome 4, the B genome, or both (Figure 6A). While the discordance among linkage group nomenclatures can often be reconciled through physical mapping of DNA sequences linked to previously genetically or physically mapped loci, our analysis highlights the critical need for the community‐wide adoption of a universal chromosome nomenclature to advance genetic studies in strawberry.

The *PF*‐associated SNPs identified in the present study and *PF*‐associated SSR and ARMS markers identified in previous studies are imperfect predictors of *PF* genotypes because of massive haplotype diversity, suppressed recombination in the *PF*‐associated LD block, and unresolved causal variation (Figures 6 and 7; Perrotte et al. 2016; Salinas et al. 2017; Honjo et al. 2016; Saiga et al. 2022). Several *PF*‐associated SNPs identified in the present study predicted the flowering habit of SDP individuals with similar accuracy (Figures 6 and 7). The A allele for the AX‐184947290 SNP (found in the Wasatch haplotype) was observed in 332 out of 379 SDP individuals classified as DN (87.6%) (Supporting Information S1). Similarly, the AX‐184947290 GG homozygote was observed in 345 out of 416 SDP individuals classified as SD (82.9%). The prediction accuracy of AX‐184947290 was only marginally greater when non‐UC individuals were trimmed from the analysis (88.5% for DN and 86.7% for SD individuals). That trend was virtually identical for several other SNPs and SNP haplotypes in the upper LD block, for example, the 33‐SNP Wasatch haplotype (W) was observed in 84.6% of the SDP individuals classified as DN (*PF*_) in the SDP population, including 55C031P001, 65C065P601, and UC DN and summer‐plant cultivars (Supporting Information S6). The obvious solution to the prediction problem is to identify the causal gene and mutation underlying *PF*; however, despite extensive previous research, the causal variant remains elusive.

Studies beyond those presented here are needed to understand how runnerless and near‐runnerless phenotypes affect yield. We hypothesized that runnering and fruit yield are negatively genetically correlated and that fruit yields can be maximized by minimizing runner proliferation. The pleiotropic effect of runnering on fruit yield, however, has not yet been commercially documented because runners are mechanically trimmed in production fields, thereby masking the negative pleiotropic effect of runner and daughter plant growth on flowering and fruit yield. One thing is certain, reduced runnering significantly decreases runner trimming costs for fruit growers. This has been substantiated by commercial testing of UCD Moxie, a reduced runnering DN cultivar that produces high yields of marketable fruit and requires substantially less runner trimming than typical DN cultivars (Figure 2).

Tenreira et al. (2017) showed that the runnerless phenotype of the *F. vesca* cultivar Yellow Wonder was caused by “a deletion mutation in a gene coding for a GA20‐oxidase enzyme needed for GA biosynthesis.” Caruana et al. (2018) induced a recessive mutation (*suppressor of runnerless‐1* [ *srl‐1*]) in Yellow Wonder that eliminated the runnerless phenotype. The *srl‐1* phenotype was subsequently found to be caused by a point mutation (G → A) that created a stop codon in *FveRGA1*, *FveRGA1*, which encodes a *DELLA* protein. They showed that the mutation affects the relative expression of *FveRGA1* in leaves and concluded “that *FveRGA1* likely represses GA signaling in multiple tissues and developmental stages” and that *FveRGA1* plays an important role in “regulating axillary meristem identity in strawberry” (Caruana et al., 2018). We originally hypothesized that the runnerless phenotype of 55C032P001 might be caused by a single gene mutation, for example, by an *RGA1* mutation; however, our results did not support that hypothesis.

While the pleiotropic effect of the *PF* locus on runnering was predictable and anticipated (Gaston et al., 2013; Hossain et al., 2019), the absence of large‐effect loci for runnering was not. The identification of single gene mutations with large effects on runnering in future studies seems plausible, particularly since *RGA1* and other genes affecting flowering and runnering have been identified and others are bound to emerge (Caruana et al., 2018; Hytönen & Kurokura, 2020; Koskela et al., 2016). We identified a homolog of one of the more promising candidate genes (*RGA1*) in the fringe of the *PF* LD block (*RGA1‐4B*; Fxa4Bg103085; bp 28,885,347–28,887,164; Figure S2). While intriguing, SNPs in LD with *RGA1‐4B* were 1.59 Mb downstream of *PF* (27,294,311) and only very weakly associated with runner count. Whether natural *RGA1‐4B* allelic variants affect runnering remains unknown. This gene, however, is an interesting target for modifying runner growth characteristics by genetic engineering or gene editing (Caruana et al., 2018).

Strawberry production could migrate, at least partly, toward seed‐propagated cultivars where runners are unnecessary. This would necessitate significant changes in current breeding and nursery production practices; specifically, a shift from the clonal propagation of hybrid individuals between outbred (partially inbred) parents to seed propagation of single‐cross hybrids between inbred parents, the formation and exploitation of heterotic groups, the implementation of inbred–hybrid breeding schemes, and the development of a hybrid seed production system (Feldmann, Pincot, Seymour, et al., 2024). Seed propagation of hybrid individuals through apomixis could be even more game‐changing (Conner & Ozias‐Akins, 2017; Mahlandt et al., 2023; Underwood & Mercier, 2022), eliminate the need for implementing resource‐intensive inbred‐hybrid breeding schemes, and facilitate less resource‐intensive within family selection schemes that have produced substantial genetic gains and can directly exploit elite genetics without inbred line development (Feldmann, Pincot, Seymour, et al., 2024; Feldmann, Pincot, Cole, et al., 2024).

## AUTHOR CONTRIBUTIONS

**Hillel Brukental**: Conceptualization; Data curation; formal analysis; investigation; methodology; Project administration; visualization; writing—original draft; writing—review and editing. **Marta L. Bjornson**: Conceptualization; data curation; formal analysis; supervision; validation; visualization; writing—review and editing. **Dominique D.A. Pincot**: Conceptualization; Data curation; methodology; Resources; writing—review and editing. **Michael A. Hardigan**: Conceptualization; Data curation; formal analysis; methodology; Resources; writing—review and editing. **Sadikshya Sharma**: Data curation; formal analysis; writing—review and editing. **Nicolas P. Jiménez**: Data curation; Formal analysis; Resources; writing—review and editing. **Randi A. Famula**: Conceptualization; Data curation;project administration; resources; supervision; writing—review and editing. **Cindy M. López**: Data curation; investigation; resources; writing—review and editing. **Glenn S. Cole**: Conceptualization; data curation; investigation; Formal analysis; Funding acquisition; methodology; Supervision; project administration; writing—review and editing. **Mitchell J. Feldmann**: data curation; formal analysis; funding acquisition; investigation; methodology; project administration; supervision; validation; visualization; writing—original draft; writingreview and editing. **Steven J. Knapp**: Conceptualization; Data curation; formal analysis; funding acquisition; investigation; methodology; project administration; resources; supervision; visualization; writingreview and editing.

## CONFLICT OF INTEREST STATEMENT

The authors declare no conflicts of interest.

## SUPPORTING INFORMATION

The phenotypic and genotypic data generated in this study have been deposited at Zenodo and are freely available (https://doi.org/10.5281/zenodo.13937028).

## Supporting information




**Supplemental File S1**. Origin years, species, pedigrees, flowering habit classifications, runner score phenotypic means, and AX‐184947290 SNP genotypes for n=932 octoploid strawberry diversity panel (SDP) individuals. The SDP included 15 *F. chiloensis*, 24 *F. virginiana*, and 893 *F*. × *ananassa* clonally propagated individuals. SDP individuals were classified as short‐day (SD = 0) or day‐neutral (DN = 1). The runner scores of SDP individuals were recorded on clonally propagated plants in Winters, CA using an ordinal scale, where 1 = runnerless, 2 = weak runnering, 3 = intermediate runnering, 4 = strong runnering, and 5 = extreme runnering. The runner score estimated marginal means (EMMs) of SDP individuals were estimated from r=1.92 observations, where r is the harmonic mean number of observations per individual (the data were unbalanced and some individuals were observed only once). Tabs 2 and 3 present the unique crosses (554) and unique parents (489) of the 932 SDP individuals.
**Supplemental File S2**. The physical positions of 50K and 850K Axiom array SNPs corroborated by genetic mapping. SNPs were physically anchored to the ‘Camarosa’ (FaCA1; Edger et al. (2019); https://phytozome‐next.jgi.doe.gov/info/Fxananassa_v1_0_a1) and ‘UCD Royal Royce’ (FaRR1; https://phytozome‐next.jgi.doe.gov/info/FxananassaRoyalRoyce_v1_0) genomes in silico. Chromosomes were numbered using the nomenclature of Hardigan et al. (2020) and cross‐referenced to the nomenclature of Edger et al. (2019). This database includes the physical positions of SNPs identified by BLAST in the ‘Camarosa’ and ‘Royal Royce’ genomes, DNA sequences of the SNP probes, chromosome assignments and physical positions of SNPs corroborated by genetic mapping, and associated information.
**Supplemental File S3**. A Rosetta stone for cross‐referencing linkage group and chromosome nomenclatures in octoploid strawberry. This database includes several previously published chromosome nomenclatures (Edger et al., 2019; Hardigan et al., 2020; Tennessen et al., 2014) and the chromosome nomenclature proposed by Session and Rokhsar (2023).
**Supplemental File S4**. Pedigrees, runner count phenotypes, and AX‐184947290 SNP genotypes for n=87 seed‐propagated 17C321P015 × 55C032P001 F_2_ and n=183 seed‐propagated 17C321P015 × ‘UCD Royal Royce’ F_2_ progeny observed March 26, April 9, April 22, May 6, May 20, June 3, and July 6, 2023 in Davis, CA (the respective photoperiods on those dates were 12.3, 13.0, 13.3, 14.1, 14.3, 14.1, and 14.5 hr). Four F_2_ families (21S950, 21S951, 21S952, and 21S953) were developed by self‐pollinating runnerless to near‐runnerless F_1_ individuals (19A907P022, 19A907P024, 19A908P012, and 19A908P053) originating in 17C321P015 × 55C032P001 and 17C321P015 × ‘UCD Royal Royce’ full‐sib families (Table 1).
**Supplemental File S5**. Pedigrees, runner count, runner score, and inflorescence count phenotypes, and AX‐184947290 SNP genotypes for 372 seed‐propagated full‐sib progeny observed May 1, June 1, and July 1, 2024 in Winters, CA (the respective photoperiods on those dates were 13.5, 14.4, and 14.5 hr). The population included nine full‐sib families developed from crosses among 13 elite UC parents (Table 1).
**Supplemental File S6**. Haplotypes for 51 phased SNPs among 808 SDP individuals. This database includes 1,606 0,1‐coded haplotypes among 808 SDP individuals classified as either short‐day (SD = 0) or day‐neutral (DN = 1).
**Supplemental File S7**. Graphical genotypes for 50K Axiom array‐genotyped SNPs segregating between Mb 25.0 and 30.4 on chromosome 4B in 17C321P015 × 55C032P001 and 17C321P015 × ‘UCD Royal Royce’ F_2_ families. This database includes runnering phenotypes, SNP genotypes, and the ordered physical positions for 140 SNP loci among 279 individuals within four F_2_ families (21S950, 21S951, 21S952, and 21S953). The predicted associations of SNPs with *PF* alleles were used to color code cells in the graphical genotype table: peach for *pf/pf*, pale green for *PF/pf*, and dark green for *PF/PF*.
**Supplemental File S8**. Genotypes and haplotypes for 15 SNPs associated with the *PF* locus on chromosome 4B among selected samples of three individuals each from an 17C321P015 × ‘UCD Royal Royce’ F_2_ family (21S950) and two 17C321P015 × 55C032P001 F_2_ families (21S952 and 21S953). The SNPs spanned bp 26,389,744 (AX‐184068183) to bp 27,597,497 (AX‐184160850). The predicted associations of SNPs with *PF* alleles were used to color code cells in the graphical genotype table: peach for *pf/pf*, pale green for *PF/pf*, and dark green for *PF/PF*.
**Supplemental File S9**. The predicted functions of 173 annotated genes spanning bp 27,597,497 to 27,418,663 on chromosome 4B in the ‘UCD Royal Royce’ reference genome (FaRR1; https://phytozome‐next.jgi.doe.gov/info/FxananassaRoyalRoyce_v1_0).
**Supplemental Figure S1**. Manhattan plots depicting the photoperiod‐dependent effect of the *PF* locus on runner count among 270 F_2_ progeny phenotyped between March 26 and July 6 2022 in Davis, CA. F_2_ individuals were genotyped with an Axiom 50K SNP array. Manhattan plots are shown for genome‐wide association studies of phenotypes observed (A) March 26 (12.3 hr), (B) April 9 (13.0 hr), (C) April 22 (13.3 hr), (D) May 6 (14.1 hr) (E) May 20 (14.3 hr), (F) June 3 (14.1 hr), and (G) July 6 (14.5 hr) among 17C321P015 × ‘UCD Royal Royce’ and 17C321P015 × 55C032P001 F_2_ progeny.
**Supplemental Figure S2**. Synteny of *REPRESSOR OF GA1* (*RGA1*) homoeologs on chromosome 4 in the A, B, C, and D genomes of octoploid strawberry (*F*. × *ananassa*) and A genome of woodland strawberry (*F. vesca*). The ortholog identified in *F. vesca* (*FveRGA1*) by Caruana et al. (2018) is shown below *F*. × *ananassa* chromosome 4 haplotypes. Homoelogs found in the octoploid are identified by the prefix Fxa. Homology was calculated using GENESPACE (Lovell et al., 2022). The synteny plot was drawn using JCVI (Tang et al., 2024). The synteny plot was developed using genes annotated in the haplotype‐phased assemblies of the ‘UCD Royal Royce’ genome, a day‐neutral *F*. × *ananassa* cultivar (https://phytozome‐next.jgi.doe.gov/info/FxananassaRoyalRoyce_v1_0.)
