# Supplementary figures and images for: The PERPETUAL FLOWERING locus: Necessary but insufficient for genomic prediction of runnerless and other asexual reproduction phenotypes in strawberry
Source: Plant Genome. 2025 Aug 19;18(3):e70086. doi: 10.1002/tpg2.70086 (PMC12365471; doi:10.1002/tpg2.70086)

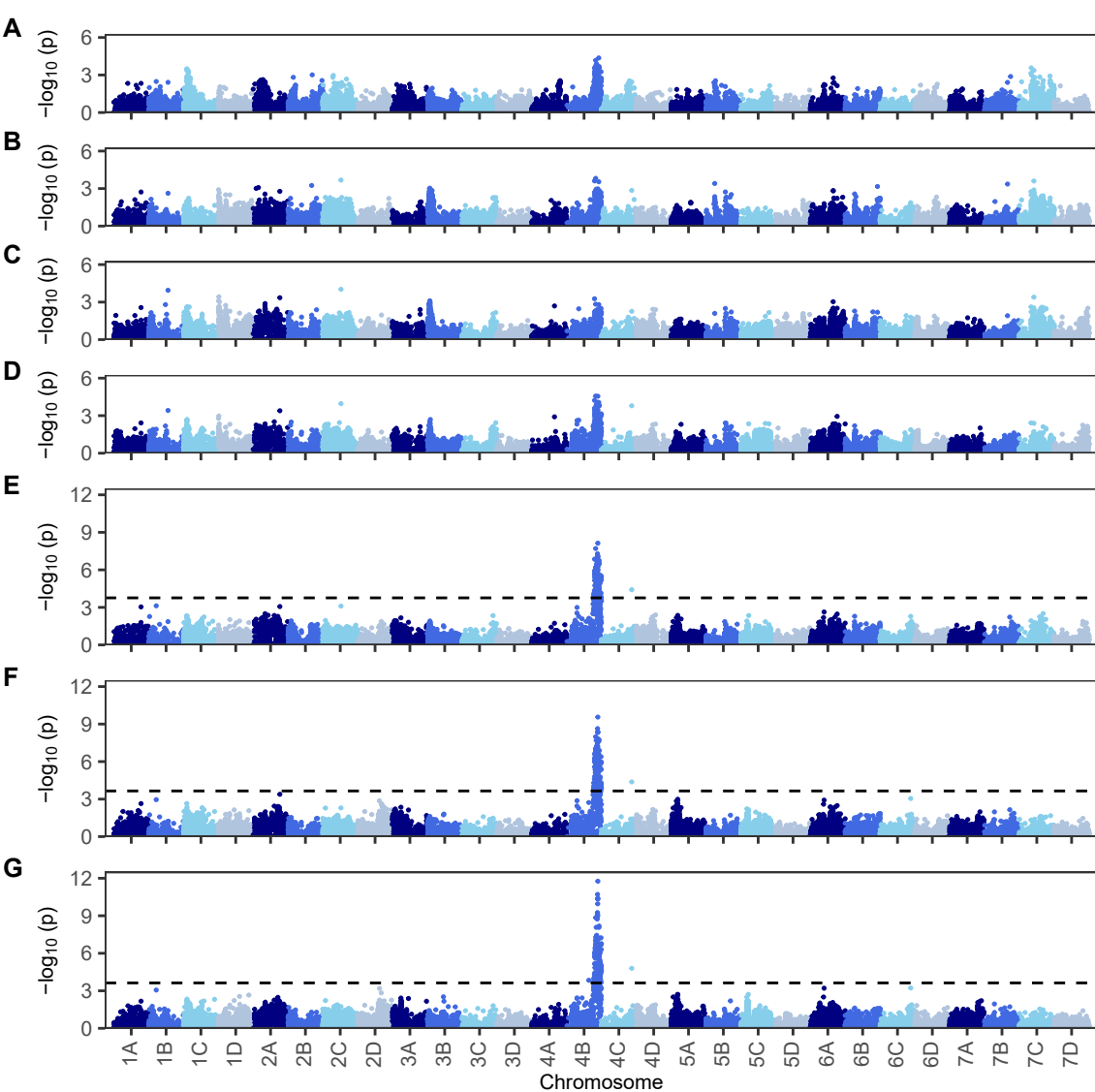

Supplement: Supplementary file 1 — Supplemental File S1. Origin years, species, pedigrees, flowering habit classifications, runner score phenotypic means, and AX‐184947290 SNP genotypes for n=932 octoploid strawberry diversity panel (SDP) individuals. The SDP included 15 F. chiloensis, 24 F. virginiana, and 893 F. × ananassa clonally propagated individuals. SDP individuals were classified as short‐day (SD = 0) or day‐neutral (DN = 1). The runner scores of SDP individuals were recorded on clonally propagated plants in Winters, CA using an ordinal scale, where 1 = runnerless, 2 = weak runnering, 3 = intermediate runnering, 4 = strong runnering, and 5 = extreme runnering. The runner score estimated marginal means (EMMs) of SDP individuals were estimated from r=1.92 observations, where r is the harmonic mean number of observations per individual (the data were unbalanced and some individuals were observed only once). Tabs 2 and 3 present the unique crosses (554) and unique parents (489) of the 932 SDP individuals. Supplemental File S2. The physical positions of 50K and 850K Axiom array SNPs corroborated by genetic mapping. SNPs were physically anchored to the ‘Camarosa’ (FaCA1; Edger et al. (2019); https://phytozome‐next.jgi.doe.gov/info/Fxananassa_v1_0_a1) and ‘UCD Royal Royce’ (FaRR1; https://phytozome‐next.jgi.doe.gov/info/FxananassaRoyalRoyce_v1_0) genomes in silico. Chromosomes were numbered using the nomenclature of Hardigan et al. (2020) and cross‐referenced to the nomenclature of Edger et al. (2019). This database includes the physical positions of SNPs identified by BLAST in the ‘Camarosa’ and ‘Royal Royce’ genomes, DNA sequences of the SNP probes, chromosome assignments and physical positions of SNPs corroborated by genetic mapping, and associated information. Supplemental File S3. A Rosetta stone for cross‐referencing linkage group and chromosome nomenclatures in octoploid strawberry. This database includes several previously published chromosome nomenclatures (Edger et al., 2019; Hardigan et [file TPG2-18-e70086-s001.zip › Submitted Supplemental Files & Figures/Supplemental Figure S1 Timeseries GWAS.pdf]

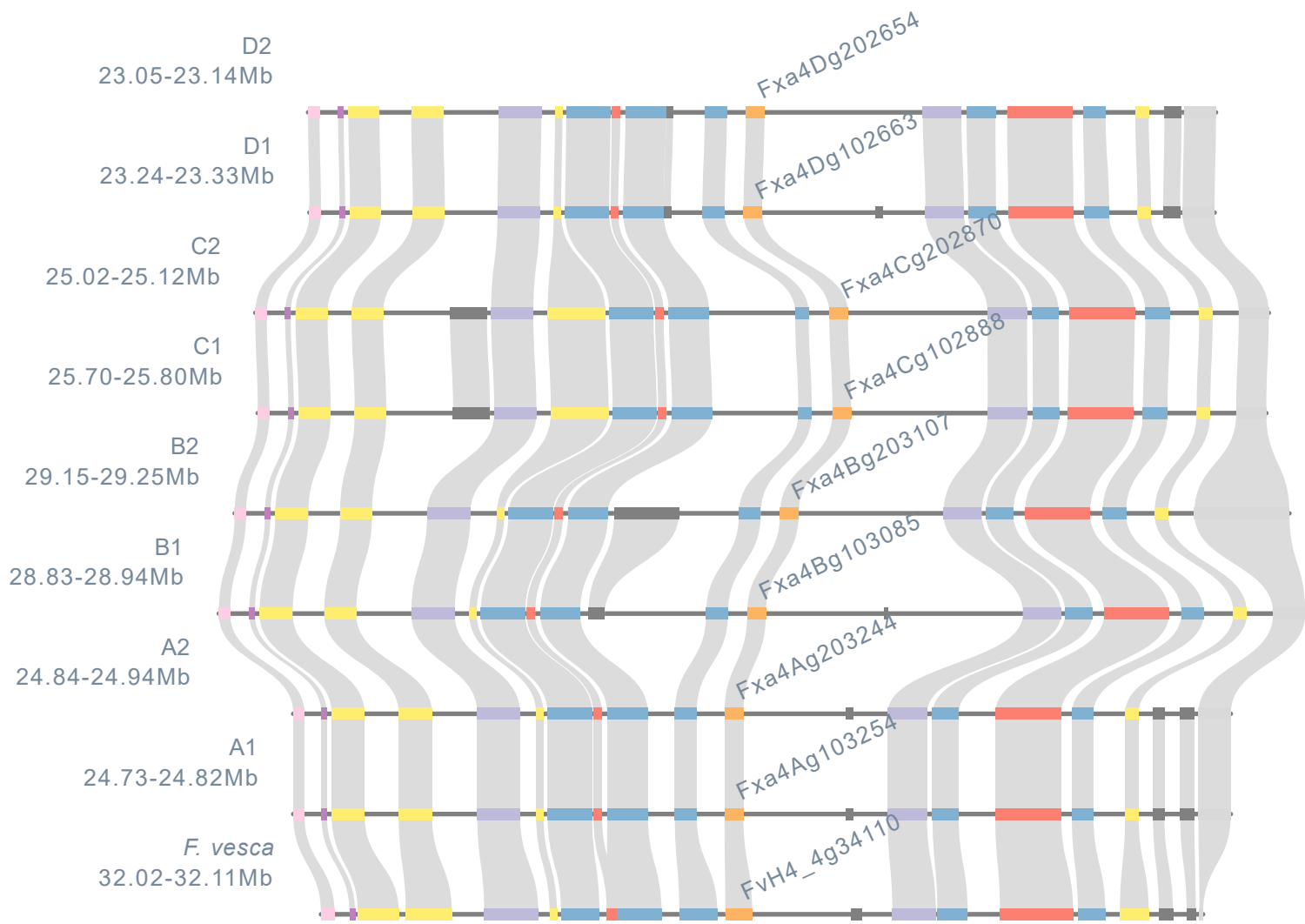

Supplement: Supplementary file 1 — Supplemental File S1. Origin years, species, pedigrees, flowering habit classifications, runner score phenotypic means, and AX‐184947290 SNP genotypes for n=932 octoploid strawberry diversity panel (SDP) individuals. The SDP included 15 F. chiloensis, 24 F. virginiana, and 893 F. × ananassa clonally propagated individuals. SDP individuals were classified as short‐day (SD = 0) or day‐neutral (DN = 1). The runner scores of SDP individuals were recorded on clonally propagated plants in Winters, CA using an ordinal scale, where 1 = runnerless, 2 = weak runnering, 3 = intermediate runnering, 4 = strong runnering, and 5 = extreme runnering. The runner score estimated marginal means (EMMs) of SDP individuals were estimated from r=1.92 observations, where r is the harmonic mean number of observations per individual (the data were unbalanced and some individuals were observed only once). Tabs 2 and 3 present the unique crosses (554) and unique parents (489) of the 932 SDP individuals. Supplemental File S2. The physical positions of 50K and 850K Axiom array SNPs corroborated by genetic mapping. SNPs were physically anchored to the ‘Camarosa’ (FaCA1; Edger et al. (2019); https://phytozome‐next.jgi.doe.gov/info/Fxananassa_v1_0_a1) and ‘UCD Royal Royce’ (FaRR1; https://phytozome‐next.jgi.doe.gov/info/FxananassaRoyalRoyce_v1_0) genomes in silico. Chromosomes were numbered using the nomenclature of Hardigan et al. (2020) and cross‐referenced to the nomenclature of Edger et al. (2019). This database includes the physical positions of SNPs identified by BLAST in the ‘Camarosa’ and ‘Royal Royce’ genomes, DNA sequences of the SNP probes, chromosome assignments and physical positions of SNPs corroborated by genetic mapping, and associated information. Supplemental File S3. A Rosetta stone for cross‐referencing linkage group and chromosome nomenclatures in octoploid strawberry. This database includes several previously published chromosome nomenclatures (Edger et al., 2019; Hardigan et [file TPG2-18-e70086-s001.zip › Submitted Supplemental Files & Figures/Supplemental Figure S2 RGA1 3-24-2025.pdf]
